# Altered *glr-1* Expression after EtOH Withdrawal Is Linked to Associative Behavior Deficits in *C. elegans*

**DOI:** 10.1523/ENEURO.0430-25.2026

**Published:** 2026-07-28

**Authors:** Katie Brandel-Ankrapp, Rachel N. Arey

**Affiliations:** ^1^Department of Neuroscience, Baylor College of Medicine, Houston, Texas 77030; ^2^Center for Precision Environmental Health, Baylor College of Medicine, Houston, Texas 77030; ^3^Department of Molecular and Cellular Biology, Baylor College of Medicine, Houston, Texas 77030

**Keywords:** AMPA receptors, *C. elegans*, CREB, ethanol, kinase, learning and memory

## Abstract

Chronic ethanol (EtOH) exposure and withdrawal are linked to worsened memory and cognitive outcomes. One target of EtOH is the glutamatergic α-amino-3-hydroxy-5-methyl-4-isoxazolepropionic acid receptor (AMPAR). Both EtOH exposure and withdrawal influences AMPAR expression, function, and broad glutamate signaling. Previous work suggests that EtOH regulation of AMPARs drives behaviors related to dependence and memory phenotypes. However, due to the complexity of the mammalian brain, it is difficult to unravel the precise mechanism by which EtOH regulates AMPARs in mammals to modify specific memory behaviors. In *Caenorhabditis elegans*, GLR-1, an AMPAR ortholog, has tightly defined expression in relatively few neurons, conserved regulatory mechanisms, and is linked to molecularly conserved associative behaviors including those disrupted by EtOH. Using an established paradigm that exposes worms to chronic EtOH and cessation (“withdrawal”), we examined the relationship between EtOH withdrawal, associative memory, and GLR-1/AMPAR regulation. We found that withdrawal from chronic EtOH disrupts intermediate-term associative memory (ITM) in wild-type worms, phenocopying loss of GLR-1 function. ITM is rescued by EtOH reintroduction, suggesting a “withdrawal-like” phenotype. Losing GLR-1 occludes the ITM deficit and EtOH-ceased wild-type phenocopy *glr-1* loss-of-function in other *glr-1*-regulated behaviors. Withdrawal broadly alters GLR-1 expression, downregulates *glr-1*, and increases CREB-mediated transcriptional activity in neurons in a JNK kinase-dependent manner. Loss of JNK and downstream transcription factor CREB prevent these EtOH withdrawal phenotypes. Overall, we have identified a transcriptional regulatory cascade driven by JNK–CREB signaling that may help neurons adapt *glr-1* signaling in response to continuous EtOH exposure and abrupt withdrawal, leading to behavior deficits.

## Significance Statement

Animals must navigate and remember their environments to survive. The neuronal processes underlying these behaviors can be disrupted by chronic EtOH. EtOH interacts with many processes and pathways in the complex mammalian nervous system. Here, we established a paradigm combining chronic EtOH exposure with associative memory conditioning in *Caenorhabditis elegans* to study the consequences of EtOH exposure and withdrawal on memory behaviors and their underlying molecular pathways. We found EtOH withdrawal leads to adaptive responses in excitatory signaling and memory deficits that depend on transcriptional regulation through a JNK–CREB signaling axis.

## Introduction

Chronic EtOH (EtOH) exposure and withdrawal are associated with persistent changes in the brain (Chandler, 2003; Abrahao et al., 2017; Hedayati-Moghadam et al., 2024; Popova et al., 2024; Lovinger and Roberto, 2025) that affect behavior, including memory (Davies et al., 2004; Lee et al., 2008, J. Lee et al., 2009; Burnett et al., 2016; Mira et al., 2019; Lindsay et al., 2022; Rose et al., 2022; Larnerd et al., 2023). Chronic EtOH exposure is linked to worsened cognitive outcomes in mammals (Farr et al., 2005; Fernandez et al., 2017; Hoffman et al., 2019; Nunes et al., 2019; Kipp et al., 2021; Sun et al., 2023), including in humans (Ambrose et al., 2001; Zinn et al., 2004; Pitel et al., 2007a,b; Noël et al., 2012; Sabia et al., 2018; Sullivan and Pfefferbaum, 2019). EtOH withdrawal also disrupts behavior (Stephens et al., 2005; Läck et al., 2007; Cannizzaro et al., 2019; Cheng et al., 2025, preprint; Qiao et al., 2018). To better understand these phenomena, it is imperative to identify molecular changes underlying these memory deficits. One conserved memory regulator is the ion-gated glutamate receptor family of α-amino-3-hydroxy-5-methyl-4-isoxazolepropionic acid receptor (AMPAR). Mammalian AMPARs regulate excitatory neurotransmission and drive synaptic changes critical for memory (Sanderson et al., 2008; Kessels and Malinow, 2009; Torquatto et al., 2019). Chronic EtOH and withdrawal modulate AMPAR/glutamate signaling across models (Ulrichsen et al., 1996; Thoma et al., 2011; Christian et al., 2012; Hermann et al., 2012; Mon et al., 2012; Wang et al., 2012, 2015; Ende et al., 2013; Pickering et al., 2015; Das et al., 2016; Prisciandaro et al., 2016; Gerace et al., 2019; Prisciandaro et al., 2019; Gerace et al., 2021). However, the molecular mechanisms regulating AMPAR signaling and their link to memory deficits in withdrawal remain unclear.

Barriers to addressing this question in mammalian models include heterogeneity of AMPAR-expressing neuronal subtypes within memory regulating circuits and the laboriousness of manipulating specific AMPAR-regulating pathways in the context of EtOH exposure, withdrawal, and behavioral assays. Indeed, a reductionist model with a simple nervous system, as well as conserved molecular and behavioral responses to EtOH and memory training, would be advantageous.

Decades of work have established the nematode *Caenorhabditis elegans* is a valuable tool to discover mechanisms underlying EtOH-related behaviors. *C. elegans* have a stereotyped nervous system consisting of only 302 neurons with mapped circuits (White et al., 1986; Witvliet et al., 2021). Despite this simplicity, worms exhibit conserved behavioral responses to EtOH including sensitivity, tolerance, and withdrawal (Davies et al., 2003, 2004; Thorsell, 2007; Kapfhamer et al., 2008; Mitchell et al., 2010; Bettinger and Davies, 2014; Mathies et al., 2015). EtOH withdrawal-related behaviors include deficits in navigation, food-race assays, and bordering, although memory behaviors have yet to be explored (Davies et al., 2004; Mitchell et al., 2010; Scott et al., 2017; Mathies et al., 2026).

In addition to EtOH-related behaviors, worms form molecularly conserved associative memories to various stimuli, including odors (Morrison and van der Kooy, 2001; Kano et al., 2008; Stetak et al., 2009; Kauffman et al., 2010; Stein and Murphy, 2014; Gharat et al., 2024). Olfactory learning and memory require GLR-1 (Morrison and van der Kooy, 2001; Stetak et al., 2009; Kauffman et al., 2010; Vukojevic et al., 2012; Gharat et al., 2024), the closest related ortholog to mammalian AMPAR-type subunits (Hart et al., 1995; Maricq et al., 1995; Brockie et al., 2001). While AMPARs are widely dispersed in mammals (Shen and Limon, 2021), *C. elegans* express GLR-1 in relatively few neuron types (Maricq et al., 1995) that are linked to specific behaviors, making the worm an attractive model to study mechanisms of EtOH and withdrawal-related changes in AMPAR regulation. Interestingly, 8 h of EtOH exposure during nonassociative memory training modulates GLR-1 and is associated with memory deficits in worms (Rose et al., 2022). However, it is unknown whether prolonged EtOH exposure or withdrawal impacts *C. elegans’* memory.

Here, we examined how withdrawal from chronic EtOH affects adult worm behaviors, including associative memory. First, we found EtOH withdrawal yields memory deficits that are rescued upon EtOH reintroduction, validating this EtOH withdrawal paradigm. Next, we found EtOH withdrawal in wild-type worms phenocopies loss of *glr-1* function and alters *glr-1*/GLR-1 expression as a possible adaptive response. Finally, we identified JNK and CREB as potential drivers of this adaptive response that are upstream of *glr-1* transcription and behavior deficits. Overall, this study reveals possible molecular responses to chronic EtOH and withdrawal that may produce behavior deficits.

## Materials and Methods

### *C. elegans* strains and maintenance

All strains were maintained at 20°C on 10 cm plates made from standard nematode growth medium (NGM; 3 g/L NaCl, 2.5 g/L of Bacto Peptone, 17 g/L Bacto Agar in milliQ water) or high NGM (3 g/L NaCl, 20 g/L of Bacto Peptone, 30 g/L Bacto Agar in milliQ water). After autoclaving and allowing molten agar to cool, 1 ml/L cholesterol (5 mg/ml in EtOH), 1 ml/L 1 M CaCl_2_, 1 ml/L 1 M MgSO_4_, and 25 ml/L 1 M potassium phosphate buffer, pH 6.0 (Brenner, 1974), were added to the media. Experiments were performed using NGM plates seeded with OP50 *E. coli* as the food source for *ad libitum* feeding.

Hypochlorite population synchronization was performed by collecting eggs from gravid hermaphrodites via exposure to an alkaline-bleach solution (85 ml water, 15 ml sodium hypochlorite, 5 ml 5 M NaOH), followed by repeated washing of eggs in 1 ml of M9 buffer (6 g/L Na_2_HPO_4_, 3 g/L KH_2_PO_4_, 5 g/L NaCl, and 1 ml/L 1 M MgSO_4_ in milliQ water).

### Strains

Wild types (N2 Bristol) were obtained from the Caenorhabditis Genetics Center (University of Minnesota).

Transgenic strains: CQ161 (*pCRE::GFP*) was first described in Kimura et al. (2002) and available in the Arey lab. KP1148 (*pglr-1::GLR-1::GFP*) was obtained from the CGC
Mutants: KP4 (*glr-1(n2461)* III), YT17 [*crh-1(tz2)* III], CB1393 [*daf-8(e1393)* I], KG532 [*kin-2(ce179)* X], VC1052 [*unc-43(gk452)* IV], VC8 [*jnk-1(gk7)* IV], and P1589 [*cmk-1(oy21)* IV] were obtained from the Caenorhabditis Genetics Center (University of Minnesota) and were outcrossed three times. RNA58 (*glr-1*;*crh-1*) mutants were generated by KB-A by crossing KP4 [*glr-1(n2461)*] with YT17 [*crh-1(tz2*)]; this strain was not outcrossed. RNA66 (*cmk-1;pCRE::GFP)* worms were generated by KB-A by crossing P1589 [*cmk-1(oy21)*] with CQ161*(pCRE::GFP)*. RNA67 (*jnk-1;pCRE::GFP)* worms were generated by KB-A by crossing VC8 [*jnk-1(gk7)*] with CQ161(*pCRE::GFP)*. These strains were not outcrossed.

### Internal EtOH quantification

Internal EtOH quantification was performed using an Alcohol Reagent Kit (Pointe Scientific Cat. No: 23-666-073). Sample collections were performed as previously reported (Alaimo et al., 2012), whereby 200 worms per condition were picked into a known volume of water following EtOH treatment and withdrawal. Wild-type worm volume was measured in FIJI to determine internal concentrations.

### EtOH treatment

EtOH treatment of worms was performed as previously described (J. Lee et al., 2009; Salim et al., 2022), in which EtOH was added to 10 cm NGM growth plates by pipetting EtOH onto the agar, wrapped in parafilm to prevent evaporation, and incubated at 20°C for 2 h. Worms were pipetted onto each plate and left to incubate at 20°C for 20–24 h. For the withdrawal group, worms were washed from EtOH plates after incubation and transferred onto normal NGM plates for 1 h, which has been published as sufficient time for internal EtOH concentrations to reach negligible levels (Scott et al., 2017).

### EtOH preference assay

EtOH preference assays were performed as previously described (J. Lee et al., 2009). Following exposure to water, EtOH, or EtOH withdrawal, 100–200 worms were washed with M9 and pipetted in the center of an unseeded 100 mM plate that is separated into quadrants. Quadrants on opposite ends of the plate had either EtOH or water pipetted into a 9-mm-sized well of the plate to reach a final concentration of 300 mM. Plates were sealed with parafilm, and EtOH was allowed to equilibrate in the agar for 2 h prior to the start of the assay. Thirty minutes after pipetting worms onto assay plates, worms were counted in each quadrant of the plate. The EtOH preference index was calculated as follows: {(#WormsEtOH quadrants) − (#Wormscontrol quadrants) / #totalworms}

### Baseline chemosensation assays

Chemotaxis, or chemosensation experiments, was performed based on previously published assays (Bargmann et al., 1993). In brief, assays were performed on unseeded 10 cm NGMs. Two marks were made on the back of the plate on opposite sides of the plate, ∼0.5 cm from the edge. A 1 µl of 1 M sodium azide (Thermo Fisher Scientific) was placed on both spots and allowed to dry before adding 1 µl of test odorant diluted in EtOH (for experiments in Extended Data Fig. 1-1*C*) or water (all other experiments) on one side and 95% EtOH on the other. The test odorant used was 10% butanone (v/v, Sigma-Aldrich). Worms were washed off their plates and subsequently washed three times with M9 buffer, then placed near the bottom center of the plate, equidistant between the two marks, and allowed to chemotax for an hour. Chemotaxis indices for each time-point were calculated as follows:

$$\mathrm{Chemotaxis}\;\mathrm{index}=(\#{\mathrm{worms}}_{\mathrm{odorant}}-\#{\mathrm{worms}}_{\mathrm{EtOH}})/(\mathrm{total}\#\mathrm{worms}).$$

### Positive olfactory associative memory assays

Water-treated, EtOH-treated, or withdrawal-treated worms were trained and tested for intermediate-term memory (ITM) changes as previously described (Kauffman et al., 2010, 2011; Hayden et al., 2024). Briefly, synchronized Day 2 adult worms were washed off plates with M9 buffer. Worms were then allowed to settle by gravity and washed twice more with M9 buffer to remove any bacteria. After washing, the worms were food-deprived for 1 h in M9 buffer. For 1× food-butanone pairing (mass-training) hereby called memory conditioning, food-deprived worms were transferred to 10 cm NGM conditioning plates seeded with OP50 *E. coli* bacteria and with a total of 16 µl of 10% butanone (Sigma-Aldrich) diluted in EtOH streaked on the lid in a “#” shape for 1 h. After conditioning, the trained population of worms was tested for chemotaxis to 10% butanone (in EtOH or water) and to an EtOH control using standard, previously described chemotaxis conditions (Bargmann et al., 1993). Different stages of memory were tested by measuring chemotaxis of different subpopulations of worms at different time-points at molecularly distinct stages of memory (Stein and Murphy, 2014). These stages are immediately after training (0 min, learning) or after being transferred to 10 cm NGM plates with fresh OP50 for 30 min (short-term associative memory), 1 h (intermediate-term associative memory), or 2 h (forgetting).

Chemotaxis indices for each time-point were calculated as described earlier. Performance index is the change in the chemotaxis index after training relative to the untrained chemotaxis index:

$$\mathrm{Performance}\;\mathrm{index}=(\mathrm{Chemotaxis}\;\mathrm{inde}x_{\mathrm{trained}})-\text{(}\mathrm{Chemotaxis}\;\mathrm{inde}x_{\mathrm{untrained}}).$$

The magnitude of performance decrease within a given genotype or treatment group between learning and memory time-points was calculated as follows:

$$\mathrm{Memory}\;\mathrm{performance}\;\mathrm{decay}=[\left(T_{\mathrm{Timepoint}}-T0_{\mathrm{Avg}})/(T0_{\mathrm{Avg}}\right)]\times 100.$$

### Local search assays

Following treatment of water, EtOH, or EtOH withdrawal, worms were picked onto unseeded NGM plates and allowed to acclimate for 1–3 min. Worms were video recorded for 5 min, keeping within the time frame of the local search period (Gray et al., 2005), and reversals and omega turns were manually scored. Recordings occurred over several days to ensure replication of results.

### RNA isolation, cDNA synthesis, and qRT-PCR

Worms were crushed in liquid nitrogen and added to Trizol (Thermo Fisher Scientific). RNA was isolated by manufacturer's instructions, followed by DNase treatment (Qiagen). cDNA was synthesized with an oligo dT primer and Superscript III reverse transcriptase enzyme (Thermo Fisher Scientific). cDNA was mixed with buffers, primers, SYBR green, and hot start Taq polymerase in a master mix prepared by a manufacturer (Thermo Fisher Scientific). Using a Quant Studio 7 Pro Dx Real-Time PCR System (Thermo Fisher Scientific), PCR reactions followed by a dissociation reaction determined specificity of the amplified product. Gene expression was quantified using the ΔΔCt method using *pmp-3* as a reference gene.

Primer sets were as follows:

*pmp-3* For: 5′- AGTTCCGGTTGGATTGGTCC -3′
*pmp-3* Rev: 5′- CCAGCACGATAGAAGGCGAT-3′
*glr-1* For: 5′-CTGTTGACGGGTCATCTGCT-3′
*glr-1* Rev: 5′-AACTGCACCTCCTTCGACTG-3′
*nmr-1* For: 5′-TGGCTGCGTTCCTAACACTT-3′
*nmr-1* Rev: 5′-TAGCCACAAAAACTGGCGGA-3′

### Confocal microscopy

Worms were paralyzed with fresh 4% levamisole diluted in M9 buffer and placed on a 4% agarose pad melted onto a microscope slide and secured with a coverslip. For *pCRE::GFP* experiments, imaging of Day 2 adult worms was performed on a Nikon Ti2E inverted microscope system with a W1 spinning disk confocal unit at 40× (treatment experiments) or 100× (for representative images in control experiments). An excitation wavelength of 488 nm for GFP with 50.8% laser power was used at 400 ms exposure, with a pixel size of 0.18 µm/pixel, bit depth of 16 bit, and *z*-step size of 0.6 µm. For repeating *pCRE::GFP* experiments in wild types, with the additional *jnk-1*, and *cmk-1* conditions, imaging of Day 2 adult worms was performed similarly but with 100 ms exposure and 61.9% laser power. For KP1148 experiments, imaging of Day 2 adult worms was performed on a Nikon Ti2E inverted microscope system with a W1 spinning disk confocal unit at 100×. An excitation wavelength of 488 nm for GFP with 61.9% laser power was used at 100 ms exposure, with a pixel size of 0.18 µm/pixel, bit depth of 16 bit, and *z*-step size of 0.2 µm.

### Image processing

All images were processed in FIJI to quantify fluorescence intensity and ROI detection (Schindelin et al., 2012). For segmentation of ROIs, *z*-stack images were transformed into maximum projections, set to 8 bit prior to thresholding with Otsu method, and watershed segmented to separate ROIs that were in proximity. The ROIs were collected in FIJI's Particle Analyzer plugin and projected onto SUM projections from the original *z*-stacks that were 16 bit depth for intensity quantification. Mean intensities collected from the SUM projection images were subtracted by the average mean intensity of three background areas within the worm. Because the GFP signal in negative controls is relatively dim to positive controls, representative images have enhanced brightness/contrast to see the outline of the worm. To analyze GLR-1 puncta images, a previously established protocol was used to measure fluorescence intensity and size of puncta (Hulsey-Vincent et al., 2023).

### Statistical analysis

Statistical data are reported in the main text, figures, and tables as noted. Significance threshold of *p* < 0.05 was used. The symbols *, **, ***, and **** refer to *p* < 0.05, 0.05, 0.005, and 0.0001, respectively. For the comparison of performance indices between two behavior conditions (e.g., water-treated vs EtOH-treated), Welch's *t* test was used because it does not assume equal standard deviations. For comparison of performance indices between three or more groups (e.g., wild-type vs two different EtOH exposures), one-way analyses of variance (ANOVAs) followed by Bonferroni’s post hoc tests for multiple comparisons were performed. Two-way ANOVAs or mixed models were used for evaluating effects between condition (water-treated, EtOH, withdrawal) and time-point (0, 0.5, 1, 2 h) on performance indices with a significant interaction between factors (*p* < 0.05) prompting Bonferroni’s post hoc analyses to determine differences between individual groups. All experiments were repeated on separate days with separate populations to confirm reproducibility of results. For data that were not normally distributed, nonparametric methods were used. The statistics table for all data is available in the Extended Data as well as at https://doi.org/10.5281/zenodo.20348125.

### Statistical analysis software

All statistics and code were run in GraphPad Prism 10, using standard toolboxes.

## Results

### Withdrawal from chronic EtOH, but not chronic EtOH itself, disrupts intermediate-term associative memory in wild-type worms

**Figure 1.**
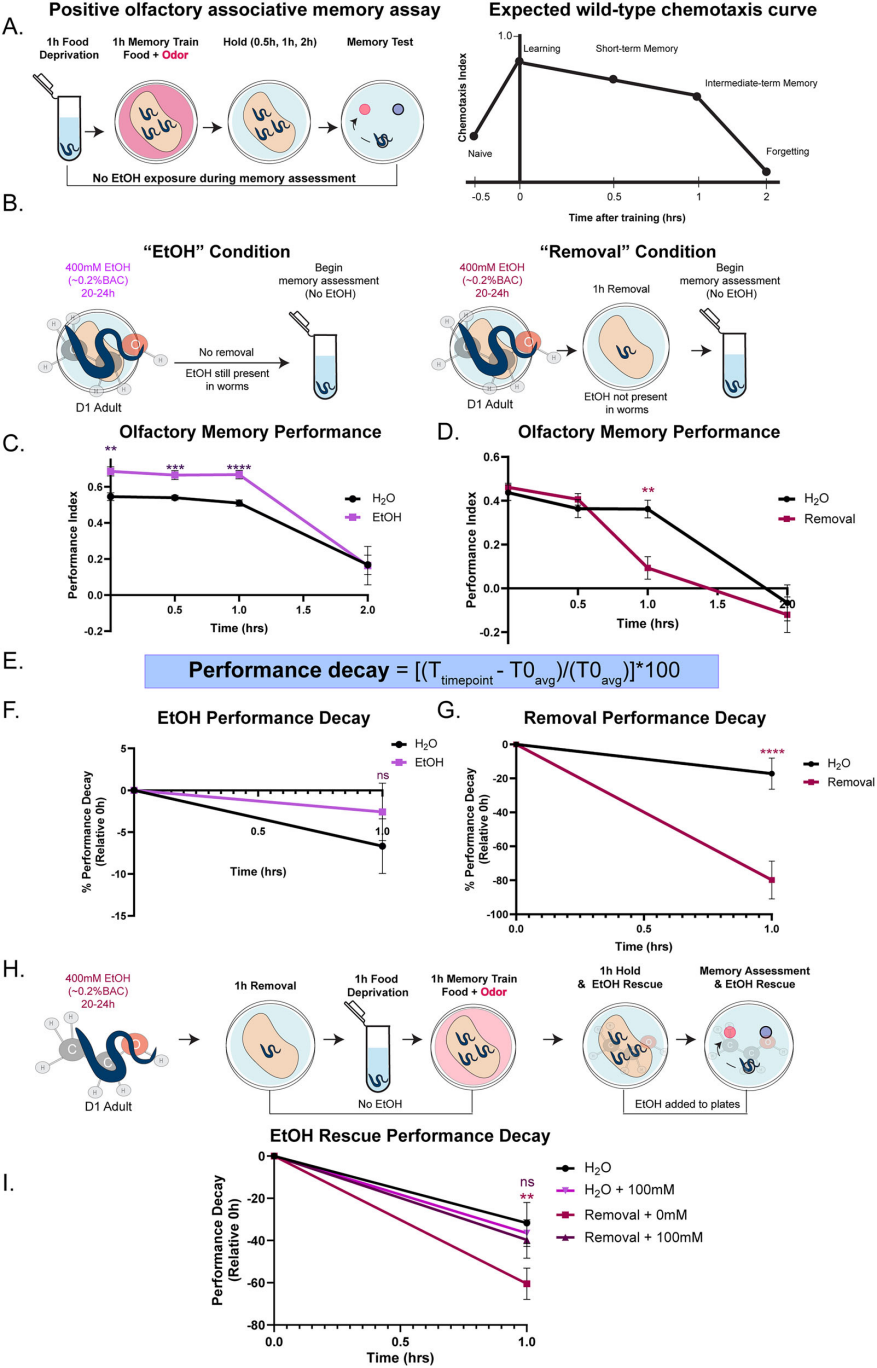
Withdrawal/removal from chronic EtOH disrupts ITM. ***A***, Schematic of experimental timeline for the positive olfactory associative memory assay. Briefly, young-adult worms are food-withdrawn for 1 h and then transferred onto food plates with 16 µl of 10% butanone in water streaked onto the top of the plate. Worms incubate on these plates for 1 h (associative memory training). Following 1 h, worms were transferred onto food plates without the odor for 30 mins, 1 h, and 2 h prior to chemotaxis assays. For the T0 time-point, worms are immediately transferred from training plates onto chemotaxis assay plates. Expected chemotaxis curves of wild-type worms for every time-point in the assay. ***B***, EtOH exposure schematic for each condition. Young-adult worms were transferred onto food and EtOH-treated plates for 20–24 h. The “EtOH” condition immediately continues to the experiment and still has detectable internal EtOH levels, while the withdrawal or “Removal” condition is transferred onto normal food plates for 1 h prior to experiments and has negligible levels of internal EtOH concentrations. To control for washes and transfers experienced by worms in EtOH withdrawal, water- and EtOH-treated worms are also washed and transferred onto their appropriate plates. ***C***, Olfactory memory performance curve of EtOH-treated and water-treated wild types across all time-points in the associative memory assay. N.s., not significant (*p* > 0.05); **p < 0.005; ***p = 0.0008; ****p < 0.0001. Error bars are SEM. *N* = 14–15 plates per condition. ***D***, Olfactory memory performance curve of 1 h Removal and water-treated wild types across all time-points in the associative memory assay. N.s., not significant, **p < 0.005. Error bars indicate SEM. *N* = 15 plates per condition. ***E***, Equation calculating performance decay, a calculation describing the difference in olfactory memory performance relative to the learning (T0) time-point. This calculation allows us to identify changes in memory performance while avoiding potential confounds in learning ability or naive chemosensation between genotypes or treatments. ***F***, Performance decay curves of water-treated and EtOH-treated wild-type worms. N.s., not significant (*p* > 0.05). Error bars are SEM. *N* = 15 plates per condition. ***G***, Performance decay curves of water-treated and removal-treated wild–type worms. ****p < 0.0001. Error bars are SEM. *N* = 15 plates per condition. ***H***, Schematic of EtOH rescue combined with the olfactory associative memory assay. Following 1 h removal from EtOH or water, worms continue through the assay as normal until the 1 h hold step. At the 1 h hold step, worms are reintroduced to EtOH by being placed on plates treated with EtOH (0 mM, 100 mM). Worms perform ITM (T60) chemotaxis on plates that are also treated with 100 mM EtOH. ***I***, Olfactory memory performance decay curve between water-treated and 1 h removal–treated wild-type retreated with 0 mM or 100 mM EtOH. N.s., not significant (*p* > 0.05); **p < 0.005. Error bars indicate SEM. *N* = 12 plates per condition. For additional behavioral data, see Extended Data Figure 1-1.

To assess the effect of EtOH withdrawal on associative learning and memory, we used a well-established positive olfactory association assay (Kauffman et al., 2010). This memory assay, which employs 1× or “massed training,” trains worms to form a short-lasting positive association with a neutral odor (10% butanone) after pairing that odorant with food for 1 h following a food deprivation period (Fig. 1*A*, left). Learning and memory are quantified by measuring worm chemotaxis to butanone at different time-points after memory training (Fig. 1*A*, right). Each learning and memory time-point is molecularly distinct and requires components needed for analogous memory stages across phyla (Kauffman et al., 2010; Stein and Murphy, 2014). We performed this assay in wild-type worms treated with water, 20–24 h 400 mM EtOH, or 20–24 h 400 mM EtOH followed by 1 h EtOH withdrawal (termed “Removal” in our figure legends), which involves washing worms from EtOH-treated plates with M9 onto normal food plates sans EtOH (Fig. 1*B*). Chronic EtOH-treated worms were transferred as well but onto EtOH-treated plates as a handling control. Water-treated worms were transferred onto water-treated plates 1 h prior to the assay. This experimental design was chosen because it would allow us to distinguish the effects of EtOH withdrawal prior to training (“Removal” condition) versus during training (“Chronic EtOH” condition).

10.1523/ENEURO.0430-25.2026.f1-1 Figure 1-1 A) Internal EtOH concentrations of day 2 adult wild-type worms across different exposure time-points, including 1h of EtOH withdrawal N = 200 worms per condition, 3-4 biological replicates per condition. B) EtOH preference indices across treatment conditions in wild-type worms. N.s., not significant (p>.05), *p<.05, ***p<.005. Error bars are SEM. N = 9 plates per condition. C) Naïve chemotaxis indices of wild-type worms with the test odorant (10% butanone) diluted in EtOH vs. water. N.s, not significant (p>.05). Error bars are SEM. N=14 plates per condition. D) Naïve chemotaxis indices of water-treated and chronic EtOH-treated wild-types to 10% butanone diluted in water. N.s., not significant (p>.05). Error bars are SEM. N=14-15 plates per condition. E) Naïve chemotaxis indices of water-treated and removal-treated wild-types to 10% butanone diluted in water. N.s., not significant (p>.05). Error bars are SEM. N=14-15 plates per condition. F) Olfactory chemotaxis curve across all time-points in water-treated and EtOH-treated wildtype worms. N.s., not significant (p>.05). Error bars are SEM. G) Olfactory chemotaxis curve across all time-points in water-treated and removal-treated wildtype worms. ***p<.0005. Error bars are SEM. N=14-15 plates per condition. Download Figure 1-1, TIF file.

We first wanted to determine that our EtOH exposure paradigm in Day 1 and Day 2 adults, which are older than in previous studies, including those that report EtOH-related behavioral impairments (Alaimo et al., 2012; Scott et al., 2017) would indeed lead to relevant internal EtOH concentrations and that 1 h of EtOH removal was sufficient time off of EtOH for internal concentrations to reach negligible levels. In agreement with previous studies, internal EtOH concentrations from 400 mM of chronic EtOH yielded a mean internal EtOH concentration of 50 mM, which equates to an intoxicating blood–EtOH concentration in humans (∼0.2%) and decreased to undetectable levels after 1 h removal (Extended Data Fig. 1-1*A*). Thus, we could examine the effects of withdrawal prior to training.

We next sought to eliminate other potential confounding variables in our paradigm. Previous studies tested chemotaxis and olfactory associative memory by providing two choices: (1) a control odorant, EtOH (weak attractant) and (2) a test odorant, typically diluted in EtOH (Bargmann et al., 1993; Kauffman et al., 2010, 2011; Stein and Murphy, 2014; Arey et al., 2018; Arey et al., 2019; Hayden et al., 2024). Interestingly, exposing worms to high external concentrations of EtOH can yield EtOH preference (J. Lee et al., 2009). Based on this, we hypothesized that our EtOH exposure protocol could lead to EtOH attraction, which could introduce a confounding variable in our measure of a positive association formed with the odor diluted in EtOH. Prior to chemotaxis experiments, we exposed worms at Day 1 of adulthood to a high external concentration of EtOH (400 mM) on their growth and maintenance plates for 20–24 h. We found worms tested immediately after 24 h of exposure had significantly increased preference to EtOH versus water-treated and EtOH withdrawal-treated worms (Extended Data Fig. 1-1*B*).

We next assessed if we could instead dilute butanone in water for our behavioral assays. We found that wild-type animals exhibit normal chemotaxis when butanone is diluted in water (Extended Data Fig. 1-1*C*); therefore, all subsequent behavior experiments were performed with water as the diluent instead of EtOH.

With conditions for assessing behavior in the context of EtOH exposure optimized, we next compared learning and memory performance after a single pairing of food with 10% butanone between young-adult wild–type worms following each treatment. Neither chronic EtOH nor EtOH withdrawal significantly altered baseline chemosensation with butanone diluted in water (Extended data Fig. 1-1*D*,*E*). Worms that were treated with chronic EtOH had significantly higher learning and memory performance indices compared with water-treated controls (Fig. 1*C*). However, their chemotaxis indices were not significantly different compared with water-treated controls (Extended data Fig. 1-1*F*), which suggests that the appearance of enhancement could be due to subtle differences in naive butanone preference, as performance indices take naive chemotaxis into account. This suggests that chronic EtOH treatment itself does not lead to deficits in positive associative learning and memory. However, worms that underwent the 1 h withdrawal period prior to the assay had a significantly lower performance and chemotaxis index than water-treated controls specifically at the ITM time-point while learning and short-term memory were spared (Fig. 1*D*, Extended data Fig. 1-1*G*).

To avoid potential confounds that could arise from differences in learning, we sought to quantify the difference in memory performance across time-points. This method, termed “performance decay,” calculates the difference from learning to subsequent memory time-points as a percentage (Fig. 1*E*), such that the magnitude of any observed changes in memory performance within a given condition or genotype can be measured. Using this quantification method, we found that EtOH-treated worms and water-treated worms had similar rates of performance decay compared with the learning time-point (Fig. 1*F*), whereas 1 h removal worms had significantly greater performance decay compared with controls (Fig. 1*G*), indicating memory impairment.

### EtOH reintroduction restores 1 h memory

To determine whether the observed ITM deficit was truly dependent on EtOH removal and was potentially withdrawal-related, we performed rescue experiments that reintroduced worms to EtOH during the memory assay. In this experiment, we reintroduced EtOH at a lower concentration than the original concentration during the 1 h hold and ITM chemotaxis steps (100 mM; Fig. 1*H*), which would allow sufficient time for EtOH to enter the worms and prevent them from withdrawing during the ITM time-point. We chose to reintroduce worms to a lower exogenous concentration of EtOH than the first exposure (∼25% of the original dose), because this strategy and a similar ratio of EtOH concentration had previously rescued behavior in withdrawn worms (Mitchell et al., 2010; Scott et al., 2017). We found reintroduction of 100 mM EtOH for the duration of the 1 h hold and ITM chemotaxis steps yielded detectable internal EtOH concentrations (Extended Data Fig. 1-1). In addition, we found EtOH-withdrawn worms that received 100 mM of EtOH reintroduction did not have significantly reduced ITM compared with water-treated worms, whereas withdrawn worms that did not receive EtOH reintroduction still showed a significant reduction in ITM (Fig. 1*I*). These experiments suggest that EtOH is entering and exiting the worms as expected and that EtOH reintroduction rescued behavior, supporting the validity of our withdrawal paradigm in testing complex behaviors. Overall, we discovered an EtOH withdrawal-dependent deficit in positive olfactory associative ITM and that these deficits are likely withdrawal-related.

### Loss of GLR-1, an AMPAR ortholog, occludes EtOH withdrawal-related behavior deficits

**Figure 2.**
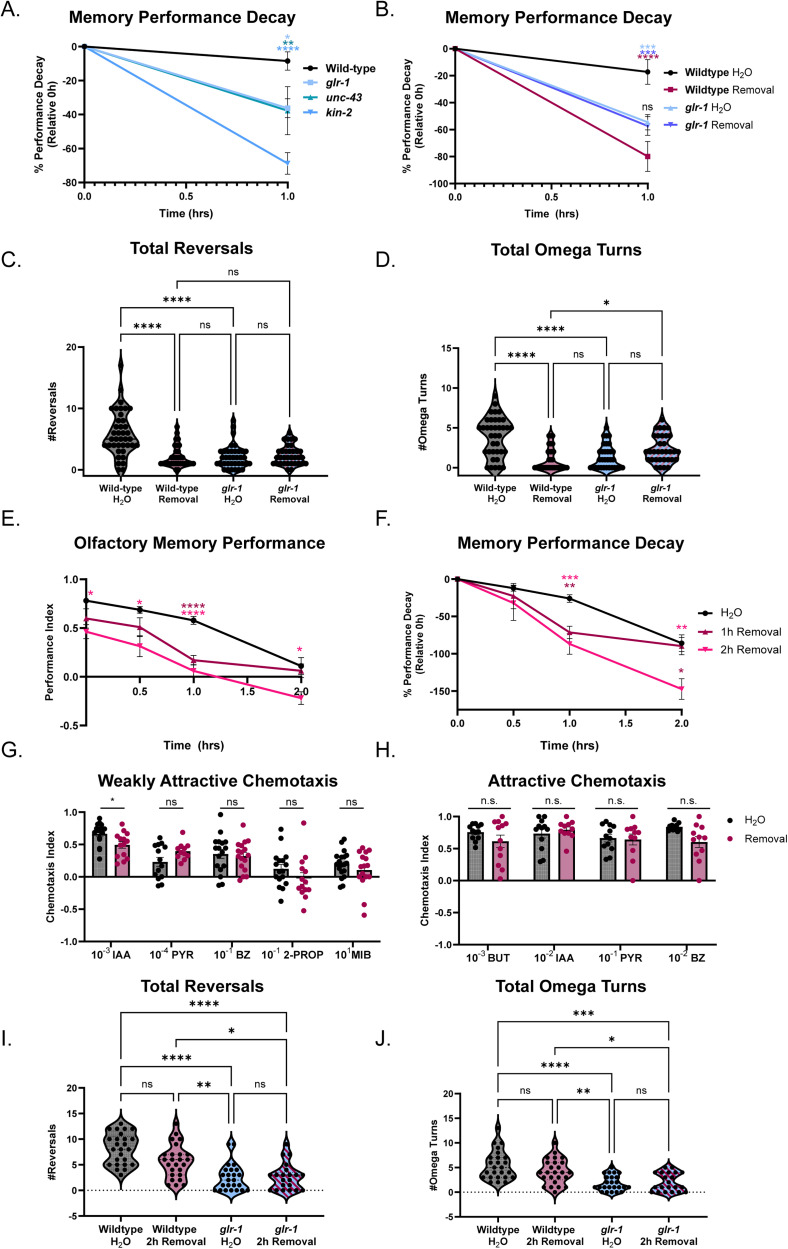
EtOH withdrawal ITM impairments are persistent, and withdrawal-treated wild–type worms phenocopy loss of *glr-1* function across multiple behaviors. ***A***, Olfactory memory performance decay curves between learning and ITM of wild-type worms, *glr-1(n2461)*, *kin-2(ce179)*, and *unc-43(gk452)* worms. *p < 0.05; **p < 0.005; ****p < 0.0001. Error bars are SEM. *N* = 15 plates per condition. ***B***, Olfactory memory performance decay curves (from T0 to T60) of wild-type worms treated with water or 1 h removal and *glr-1(n2461)* worms treated with water or 1 h removal. N.s., not significant (*p* > 0.05); ***p < 0.0005; ****p < 0.0001. Error bars are SEM. *N* = 15 plates per condition. ***C***, Reversals during local search behavior in water-treated wild–type and *glr-1(n2461)* worms and withdrawal-treated wild–type and *glr-1(n2461)* worms. N.s., not significant (*p* > 0.05); ***p < 0.0001. Error bars are SEM. *N* = 33–39 worms per condition. ***D***, Omega turns during local search behavior in water- or 1 h removal-treated wild–type and *glr-1*(n2461). N.s., not significant (*p* > 0.05); *p < 0.05; ****p < 0.0001. Error bars are SEM.*N* = 32–37 worms per condition. ***E***, Olfactory memory performance curves across all time-points in worms treated with water, 1 h removal, and 2 h removal. *p < 0.05; ****p < 0.0001. Error bars are SEM. *N* = 14–15 plates per condition. ***F***, Olfactory memory performance decay curves between T0 and T60 in worms treated with water, 1 h removal, and 2 h removal. N.s., not significant (*p* > 0.05); *p < 0.05; **p < 0.005; ***p < 0.0005. Error bars are SEM. *N* = 15 plates per condition. ***G***, Naive chemotaxis indices across a weakly attractive odorant battery in water-treated and removal-treated wild–type worms. (IAA, isoamyl alcohol; PYR, pyrazine; BZ, benzaldehyde; 2-PROP, 2-propanol; MIB, methyl-isobutyrate). N.s., not significant (*p* > 0.05); *p < 0.05. Error bars are SEM. *N* = 15–19 plates per condition per odorant. ***H***, Naive chemotaxis indices across an attractive odorant battery in water-treated and removal-treated wild–type worms (BUT, butanone; IAA, isoamyl alcohol; PYR, pyrazine; BZ, benzaldehyde). N.s., not significant (*p* > 0.05). Error bars are SEM. *N* = 8–12 plates per condition per odorant. ***I***, Reversals during local search behavior in water-treated or 2 h removal-treated wild–type and *glr-1(n2461)* worms. N.s., not significant (*p* > 0.05); *p < 0.05; **p < 0.007; ****p < 0.0001. Error bars are SEM.*N* = 21–25 worms per condition. ***J***, Omega turns during local search behavior in water-treated or 2 h removal-treated wild-type and *glr-1(n2461)* worms. N.s., not significant (*p* > 0.05); *p < 0.05; **p < 0.007; **p < 0.0005; ****p < 0.0001. Error bars are SEM. *N* = 21–25 worms per condition. For additional behavioral data, see Extended Data Figure 2-1.

The ITM deficit we observed following EtOH removal was similar to *glr-1* loss-of-function mutants. Previous studies demonstrate that learning and memory in *C. elegans* requires GLR-1 (Morrison and van der Kooy, 2001; Rose et al., 2003; Kano et al., 2008; Stetak et al., 2009; Kauffman et al., 2010; Vukojevic et al., 2012; Hadziselimovic et al., 2014; Gharat et al., 2024) which led us to hypothesize that withdrawal from chronic EtOH interacts with GLR-1 to lead to ITM deficits. We confirmed that loss of GLR-1 results in memory deficits in our assay, whereby *glr-1* mutant animals exhibit normal learning but have significantly reduced ITM when compared with wild types (Extended Data Fig. 2-1*A*,*B*). GLR-1–deficient worms do not perform worse than mutants lacking other ITM regulators, including canonical memory regulators PKA and CAMKII [*kin-2(ce179)* and *unc-43(gk452)*, respectively; Fig. 2*A*; Extended Data Fig. 2-1*A*], suggesting that *glr-1* regulates ITM without producing a floor effect, which would allow us to examine interactions between *glr-1* and EtOH withdrawal.

10.1523/ENEURO.0430-25.2026.f2-1 Figure 2-1 A) Olfactory memory performance across T0 and T60 time-points in wildtype, *glr-1*, *kin-2*, and *unc-43* mutant worms. N.s., not significant (p>.05), *p<.05, ***p<.0007, ****p<.0001. Error bars are SEM. N=15 plates per condition. B) Naïve chemotaxis in *glr-1* worms treated with water or 1h removal. N.s., not significant (p>.05). Error bars are SEM. N=14-15 plates per condition. Download Figure 2-1, TIF file.

We then evaluated the behavior of *glr-1* animals after EtOH withdrawal. We found that EtOH-removed *glr-1* mutants performed similarly to water-treated mutants at the ITM time-point and did not exhibit a change in ITM performance indices between treatments (Fig. 2*B*), nor did their naive chemosensation to 10% butanone in water significantly differ (Extended Data Fig. 2-1*B*). This contrasts with wild-type animals that exhibited a decrease in ITM performance indices after EtOH withdrawal. These results suggest an interaction between EtOH withdrawal and *glr-1*, as *glr-1* worms appear to be resistant from any further ITM deficit associated with EtOH withdrawal.

Based on our findings that EtOH withdrawal appears to phenocopy *glr-1* reduction of function and that *glr-1* mutants might exhibit resistance to EtOH withdrawal-related behaviors, we sought to further examine the potential relationship between EtOH withdrawal and *glr-1*. To do this, we compared glutamate-dependent foraging behaviors between wild-type and *glr-1* worms across water-treated and 1 h EtOH withdrawal conditions. These foraging behaviors, reversals and omega turns, are most prominent within minutes (1–12) of transferring worms from maintenance plates onto plates with no bacteria (Gray et al., 2005). Previous studies have shown loss of *glr-1* represses these behaviors (Hills et al., 2004; Juo et al., 2007; McGehee et al., 2015; Campbell et al., 2016; Bhardwaj et al., 2020; Luth et al., 2021; Park et al., 2021), whereas gain of GLR-1 activity increases these behaviors (Zheng et al., 1999; Luth et al., 2021), providing a direct behavioral readout of *glr-1* function. We found, as reported previously, untreated *glr-1* mutants performed fewer reversals and omega turns than untreated wild-type animals. Wild-type worms that received EtOH withdrawal also performed significantly fewer reversals than untreated wild types, phenocopying *glr-1* worms, while withdrawal did not significantly reduce *glr-1* mutant navigation behavior, similar to what we observed with ITM (Fig. 2*C*,*D*). Although EtOH-withdrawn *glr-1* worms showed a significant increase in omega turn frequency compared with water-treated *glr-1* worms (Fig. 2*D*), it is possible that withdrawal impacts additional regulators of omega turns (Gray et al., 2005). These results provide further evidence that EtOH withdrawal leads to reduced *glr-1* function.

### Associative behavioral deficits persist following withdrawal and are not accompanied by widespread sensory or motor dysfunction

To determine if more prolonged EtOH withdrawal resulted in similar deficits, we tested olfactory associative memory in worms treated with water, 1 h withdrawal, and 2 h withdrawal. We found that 2 h of EtOH withdrawal impairs ITM to a degree that is not significantly different from 1 h of EtOH withdrawal but is significantly lower than water-treated controls (Fig. 2*E*,*F*). Interestingly, we also found that 2 h of withdrawal led to enhanced forgetting compared with 1 h withdrawal and water treatment (Fig. 2*E*,*F*). Because memory was still impaired after prolonged EtOH withdrawal, we wanted to rule out the possibility that our EtOH exposure and withdrawal paradigm produced irreversible nervous system damage in the worms. Toward this, we tested chemosensation and navigation behaviors. First, we tested worms across a chemotaxis battery of weakly attractive and more strongly attractive odorants. We found that EtOH-removed worms had a slight but significant decrease in chemotaxis toward 0.01% isoamyl alcohol (10^−3^ IAA), which is sensed by the same neuron as butanone, the AWC. However, EtOH withdrawal did not significantly alter chemotaxis toward any other odorant in this assay, including other odorants sensed by the AWC, the AWA, and the AWB (Fig. 2*G*,*H*). These data suggest that EtOH withdrawal impairs memory independently of basic chemosensation and broad deficits in preference to weakly attractive odorants were not formed.

We next tested navigation behaviors of worms following 2 h EtOH withdrawal and found that the frequency of reversals and omega turns were no longer significantly lower than water-treated wild types (Fig. 2*I*,*J*). This suggests that while *glr-1*-regulated navigation behaviors begin to be restored after prolonged EtOH withdrawal, memory is uniquely susceptible to EtOH withdrawal-related deficits. Furthermore, these data indicate that EtOH withdrawal does not irreparably damage sensorimotor function. Rather, EtOH withdrawal appears to persistently and selectively impair memory.

Overall, our data provide evidence that EtOH withdrawal impacts behavior in a way that is akin to loss of *glr-1* function. Early (1 h) EtOH withdrawal impairs multiple behaviors regulated by *glr-1*, including ITM. After more protracted (2 h) EtOH withdrawal, navigation behaviors are restored, but the ITM impairment persists alongside enhanced forgetting. This led us to hypothesize that *glr-1* is being downregulated before memory training commences, and this prevents memory consolidation. While *glr-1* may be reestablished hours later, permitting navigation behaviors, this restoration is too far removed from memory training to permit ITM.

### Acute EtOH withdrawal leads to *glr-1* downregulation

**Figure 3.**
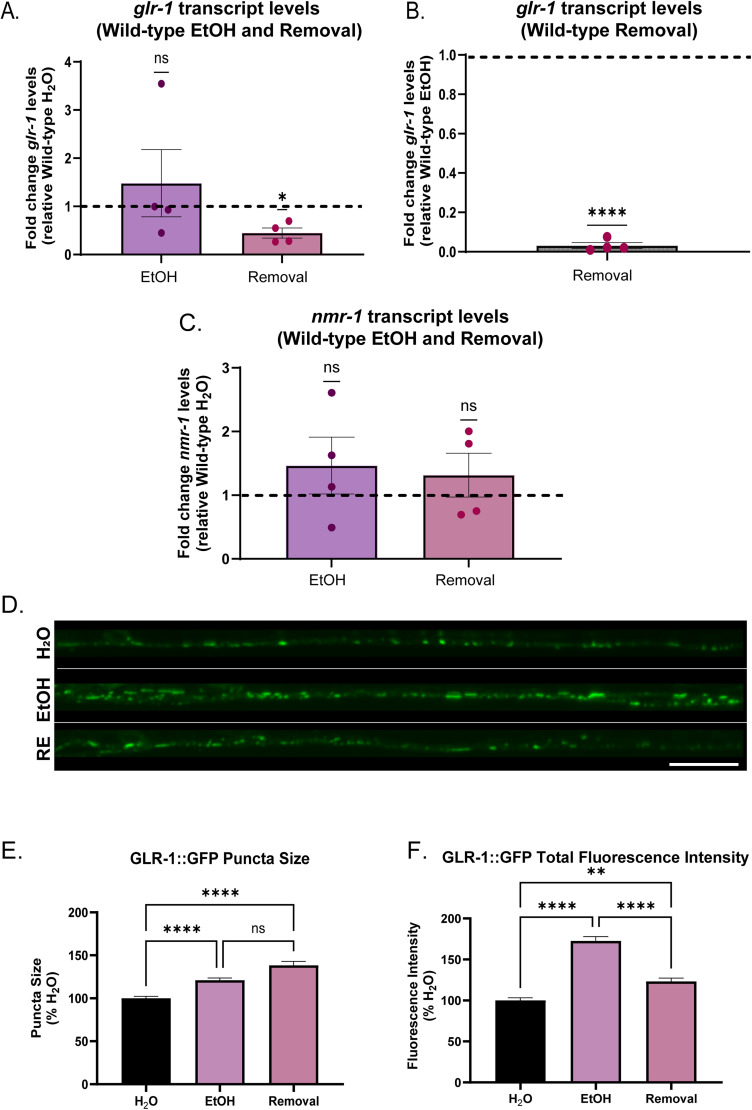
Withdrawal from chronic EtOH downregulates *glr-1* and alters GLR-1 expression in the ventral nerve cord. ***A***, Fold change in *glr-1* transcript levels between EtOH- and withdrawal-treated wild types relative to water-treated wild types. N.s., not significant (*p* > 0.05); *p < 0.05. Error bars are SEM. *N* = 4 biological replicates. Dashed lines indicated fold change of 1. ***B***, Fold change in *glr-1* transcript levels in removal-treated wild types relative to EtOH-treated wild types; ***p < 0.0001. Error bars are SEM. *N* = 4 biological replicates. Dashed lines indicated fold change of 1. ***C***, Fold change in *nmr-1* transcript levels between EtOH and withdrawal-treated wild types relative to water-treated wild types. N.s., not significant (*p* > 0.05). Error bars are SEM. *N* = 4 biological replicates. Dashed lines indicated fold change of 1. ***D***, Representative confocal images of GLR-1 in the ventral nerve cord in KP1148 (*p-glr-1*::GLR-1::GFP) worms following water, chronic EtOH, and 1 h removal treatment. H_2_O, water-treated; EtOH, chronic EtOH-treated; RE, EtOH withdrawal-treated. Scale bar, 10 µM. ***E***, Size of GLR-1 puncta in the ventral nerve cord in KP1148 (*p-glr-1*::GLR-1::GFP) following water, EtOH, and 1 h removal treatment. N.s., not significant (*p* > 0.05); ***p < 0.0001. Error bars are SEM. *N* = 721–821 puncta. ***F***, Fluorescence intensity of GLR-1 puncta in the ventral nerve cord in KP1148 (*p-glr-1*::GLR-1::GFP) following water, EtOH, and 1 h removal treatment. **p < 0.005; ****p < 0.0001. Error bars are SEM. *N* = 723–820 puncta.

Based on our behavior data, we hypothesized that 1 h withdrawal from chronic EtOH would lead to decreased *glr-1* expression prior to memory training. We first evaluated this at the transcript level using quantitative real-time PCR (qPCR) in wild-type worms receiving either water, chronic EtOH, or EtOH withdrawal (1 h). While *glr-1* transcript levels are unchanged relative to water controls in worms that were still on EtOH at the time of collection, 1 h withdrawal resulted in a significant downregulation of *glr-1* transcript levels compared with other treatments (Fig. 3*A*,*B*). To assess if this downregulation was specific to *glr-1* glutamate receptors, we also measured transcript levels of another type of ionotropic glutamate receptor, *nmr-1*, which is orthologous to mammalian *N*-methyl-d-aspartate (NMDA) receptor proteins. NMDA receptors are critical for plasticity and memory (Malenka and Nicoll, 1999) and are EtOH targets (Kumari and Ticku, 2000; Y. Wang et al., 2007; Nagy, 2008; Morisot and Ron, 2017). Interestingly, we found that *nmr-1* transcript levels were not significantly different compared with water-treated wild types in all exposure conditions measured (Fig. 3*C*). These data suggest that a general downregulation of ionotropic glutamate receptors in nematodes does not occur after EtOH withdrawal, suggesting that EtOH withdrawal may specifically impact *glr-1* transcription, rather than widely repress ionotropic glutamate receptor transcription.

We next investigated if downregulated mRNA levels of *glr-1* could correspond to changes in synaptic GLR-1 protein. Toward this, we utilized the KP1148 strain (*pglr-1*::GLR-1::GFP), a translational reporter of GLR-1 that allows for visualization and analysis of GLR-1 puncta along the ventral nerve cord (VNC). Analysis of VNC GLR-1 puncta has been previously used to measure changes in GLR-1 expression in response to memory training (Rose et al., 2003; Stetak et al., 2009) and has been correlated as a functional readout with changes in *glr-1* mRNA expression in the context of EtOH exposure and memory training (Rose et al., 2022). Thus, we asked if GLR-1 expression was altered by chronic EtOH exposure or EtOH withdrawal. Using a previously developed method for analyzing puncta in Fiji (Hulsey-Vincent et al., 2023), we found that, compared with water-treated worms, GLR-1 puncta of worms chronically exposed to EtOH had significantly higher fluorescence intensity and were significantly larger in size. Following EtOH withdrawal, puncta size remained large, but fluorescence intensity significantly decreased compared with chronic EtOH-treated worms (Fig. 3*D*–*F*). Our data suggest that the expression of GLR-1 in the VNC is dynamically regulated across chronic EtOH exposure and EtOH withdrawal and that changes in *glr-1* could be linked to changes in protein levels. Our results are similar to those in previous studies across multiple models that have observed increased synaptic AMPAR expression following chronic EtOH exposure or early withdrawal that is linked to adaptive increases in cell excitability and altered behavior (Christian et al., 2012; Wang et al., 2012, 2015; Faccidomo et al., 2021).

### *glr-1* downregulation and GLR-1-regulated behavior deficits in EtOH withdrawal are CREB-dependent

**Figure 4.**
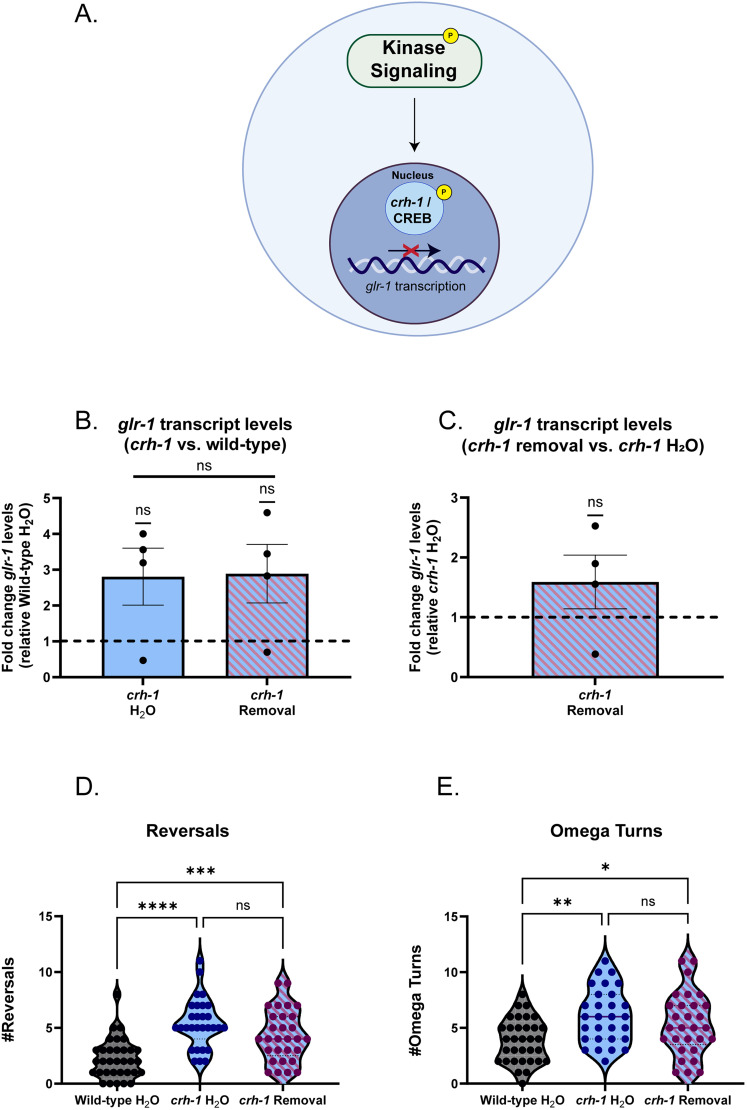
Changes in *glr-1* expression and behavior in EtOH withdrawal may be CREB-dependent. ***A***, Schematic of kinase-mediated CREB signaling pathway known to transcriptionally regulate *glr-1*. ***B***, Relative fold change of *glr-1* mRNA levels in *crh-1* worms treated with water or EtOH removal relative to wild-type controls. N.s., not significant (*p* > 0.05). Error bars are SEM. *N* = 4 biological replicates. Dashed lines indicated fold change of 1. ***C***, Relative fold change of *glr-1* mRNA levels in *crh-1* worms following 1 h EtOH removal relative to water-treated *crh-1* worms. N.s., not significant. Error bars are SEM. *N* = 4 biological replicates. Dashed lines indicated fold change of 1. ***D***, Total number of reversals during local search behavior in wild-type, *crh-1* worms after water or withdrawal treatment. N.s., not significant (*p* > 0.05); *p < 0.05; **p < 0.005. Error bars are SEM. *N* = 29–33 worms per condition. ***E***, Total number of omega turns during local search behavior in wild-type and *crh-1* worms after water treatment or EtOH withdrawal. N.s., not significant; ***p = 0.0005; ****p < 0.0001. Errors bars are SEM. *N* = 29–33 worms per condition. For additional gene expression and behavioral data see Extended Data Figure 4-1.

Because the earliest change in *glr-1* we observed was at the transcriptional level, we next sought to identify the molecular pathway underlying EtOH withdrawal-related *glr-1* downregulation. We therefore turned to established transcriptional regulators of *glr-1* that are known to broadly respond to changes in neuronal excitability and could potentially modulate an adaptive response in *glr-1* levels. Previous studies have uncovered negative transcriptional regulators of *glr-1*, including those that are downstream of conserved molecular pathways in neurons such as CMK-1/CAMKIV and DAF-7/TGF-β (McGehee et al., 2015; Moss et al., 2016), which act through downstream transcription factor CRH-1/CREB and RSMAD/DAF-8, respectively (Fig. 4*A*; Extended Data Fig. 4-1*A*). Interestingly, alterations in both neuronal TGF-β and CREB expression or activity have been documented in EtOH treatment in other models (Pandey et al., 2001; Bison and Crews, 2003; J. Wang et al., 2007; Shibasaki et al., 2011; Kurokawa et al., 2013; Huang et al., 2015; Qiao et al., 2018; McClintick et al., 2020). Furthermore, CREB activation and CREB-mediated transcription is activated in response to stress-induced changes in glutamatergic signaling in the nervous system, including in larval *C. elegans* (Liu et al., 2004; Kitagawa, 2007; Feldmann et al., 2019; Mendelowitz, 2022), playing a role in neuroprotection against changes in neuronal excitability. TGF-β may play a role in this as well and is known to regulate *glr-1* in the worm (Prehn et al., 1993; Prehn and Miller, 1996; Boche et al., 2003; McGehee et al., 2015). Therefore, these pathways were attractive candidates for playing a role in *glr-1* downregulation during EtOH withdrawal.

To test the role of these transcriptional regulators in *glr-1* expression during EtOH withdrawal, we performed qPCR in either *crh-1* or *daf-8* mutant backgrounds following treatment with water or EtOH withdrawal. We hypothesized that if either *daf-8* or *crh-1* is involved in *glr-1* downregulation during EtOH withdrawal, then removing its ability to repress *glr-1* via genetic ablation would prevent withdrawal-related *glr-1* downregulation. When we assessed *glr-1* levels in *crh-1* mutants, we did not observe increased *glr-1* transcript levels as previously reported (Moss et al., 2016; Fig. 4*B*). This discrepancy could be due to differences in life stage, as we collected synchronized Day 2 adults for all experiments instead of mixed populations, or at the L4 stage that were used in previous work (Moss et al., 2016) or because we measured endogenous *glr-1* levels instead of a transcriptional *glr-1* reporter. However, we did find that *crh-1* worms did not exhibit a significant decrease in *glr-1* expression after EtOH withdrawal (Fig. 4*C*). These results are consistent with *crh-1* being a negative regulator of *glr-1* as previously reported in Moss et al. (2016), albeit in a context-dependent manner in adulthood. These results also suggested that CRH-1/CREB is required for withdrawal-related downregulation of *glr-1* transcription.

10.1523/ENEURO.0430-25.2026.f4-1 Figure 4-1 A) Pathway describing *glr-1* transcriptional regulation via DAF-7/TGF-β. B) Relative fold change of *glr-1* mRNA levels in *daf-8* background relative to wild-type controls N.s. not significant (p>.05), *p<.05. Error bars are SEM. N=4-5 Biological replicates. Dashed lines indicated fold change of 1. C) Relative fold change of *glr-1* levels in *daf-8* treated with 1h removal vs. water-treated *daf-8* worms. *p<.05. Error bars are SEM. N=4 Biological replicates. Dashed lines indicated fold change of 1. D) Olfactory chemotaxis curves of wildtypes and *crh-1*(tz2) worms. **p<.005. Error bars are SEM. N=9-10 plates per condition. E) Olfactory chemotaxis index of wildtypes, *glr-1*(n2461), *crh-1*(tz2), and *glr-1;crh-1* worms at T30 (STM) time-point. N.s., not significant (p>.05), **p<.005. Error bars are SEM. N=10 plates per condition. Download Figure 4-1, TIF file.

When we examined *daf-8* mutants, we found that in accordance with previous findings, they had significantly higher *glr-1* transcript levels compared with wild-type worms (Extended Data Fig. 4-1*B*; McGehee et al., 2015). However, following EtOH withdrawal, *daf-8* worms no longer had significantly higher *glr-1* levels relative to wild types. Direct comparison of *daf-8* water-treated worms and *daf-8* EtOH-withdrawn worms was not significant, although the fold change of *glr-1* transcript levels was significantly lower in *daf-8* worms after EtOH withdrawal relative to *daf-8* water-treated worms (Extended Data Fig. 4-1*C*). These results indicate that the downregulation in *glr-1* following EtOH withdrawal was likely not dependent on *daf-8*. Overall, these results suggest that a specific molecular pathway involving *crh-1* or CRH-1-mediated transcription is required for *glr-1* downregulation during EtOH withdrawal in *C. elegans*.

Because losing *crh-1* is protective against *glr-1* downregulation, we hypothesized that in EtOH withdrawal *crh-1* mutants would not exhibit behavior deficits that wild-type animals exhibit in our previously tested *glr-1*–dependent behaviors. However, we found that *crh-1* worms alone exhibit a severe ITM deficit, similar to the extremely poor-performing *kin-2* mutants (Fig. 2*A*), when compared with wild-type worms in our positive olfactory associative memory assay (Extended Data Fig. 4-1*D*). As a widely expressed transcription factor, CREB plays a significant role in regulation of neuronal function, and this behavioral deficit is likely not solely due to *glr-1*/AMPA receptors. To test the combined roles of CREB and GLR-1 on ITM, we created double mutants of both genes. When we tested the double mutants in our assay, we found losing both *crh-1* and *glr-1* led to significant deficits beginning at 30 min after training (short-term associative memory; Extended Data Fig. 4-1*E*) compared with wild-type worms and single mutants of *crh-1* and *glr-1*. This led us to conclude that the memory deficits observed in *crh-1* mutants are at least partially independent of *glr-1*, which combined with their severity would confound our ability to interpret the effects of CRH-1/CREB's role in EtOH withdrawal-mediated memory deficits. We therefore examined if *crh-1* mutants exhibited resistance to withdrawal-mediated alterations in local search behaviors, which are a more *glr-1*–specific phenotype. We first found that *crh-1* mutants performed reversals and omega turns significantly more frequently than wild-type worms (Fig. 4*D*,*E*), which could suggest increased glutamate receptor function. We also found that EtOH withdrawal had no detectable effect on either reversals or omega turns of *crh-1* mutants (Fig. 4*D*,*E*) compared with water-treated *crh-1* worms, indicating that losing *crh-1* is protective against loss of *glr-1* phenotypes. Together these findings suggest that *crh-1* negatively regulates *glr-1* in the context of EtOH withdrawal and may be linked to deficits in *glr-1*-dependent behaviors in EtOH withdrawal.

### CREB-mediated transcription in neurons increases after EtOH withdrawal

**Figure 5.**
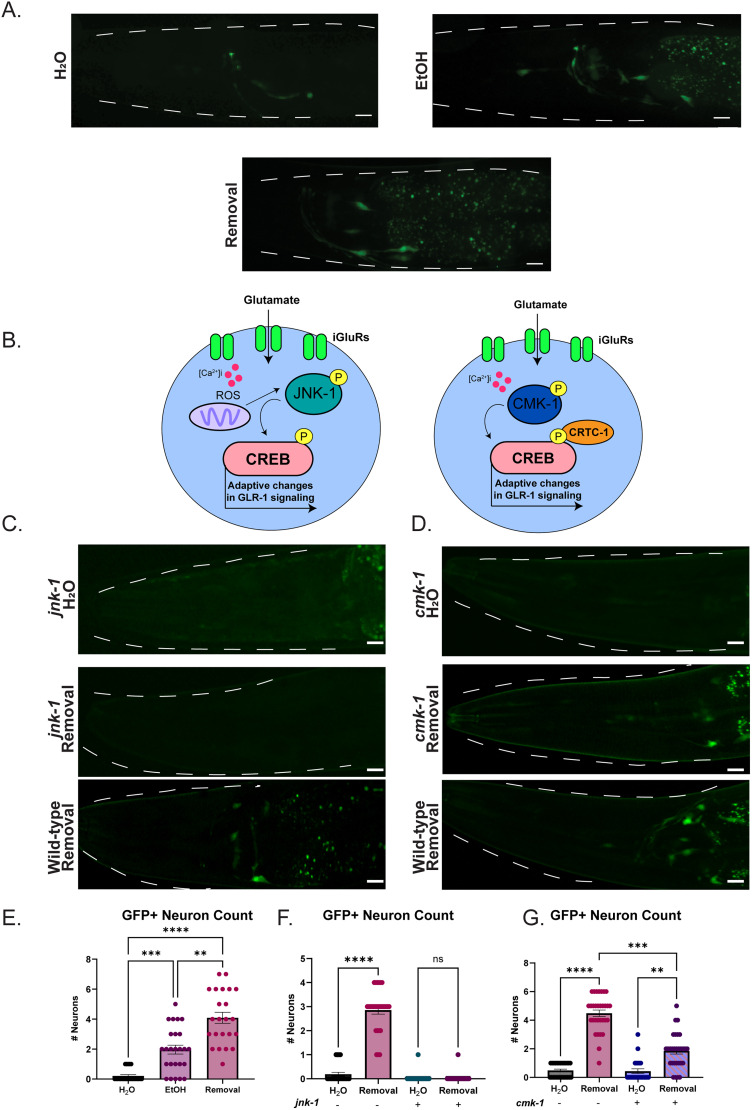
CREB-mediated transcription is increased after EtOH withdrawal in a JNK-dependent manner. ***A***, Representative images of *pCRE::GFP* worms across water, EtOH, and 1 h removal exposure conditions. Scale bar, 10 µM. ***B***, Candidate pathways downstream of CMK-1 and JNK-1 known to increase CREB activity in response to glutamate signaling or oxidative stress. ***C***, Representative images of *jnk-*1 × *pCRE::GFP* worms treated with water and 1 h removal and *pCRE::GFP* treated with 1 h removal. Scale bar, 10 µM. ***D***, Representative images of *cmk-1* × *pCRE::GFP* worms treated with water and 1 h removal and *pCRE::GFP* worms treated with 1 h removal. ***E***, Number of GFP^+^ neurons detected in wild-type *pCRE::GFP* worms across all exposure conditions. **p = 0.0062; ***p = 0.0006;****p < 0.0001. Error bars indicate SEM. *N* = 23–25 worms/condition. ***F***, Number of GFP^+^ neurons detected in *pCRE::GFP* worms in a wild-type or *jnk-1* background treated with water and 1 h removal and *pCRE::GFP* treated with 1 h removal. N.s., not significant (*p* > 0.05); ***p < 0.0001. Error bars are SEM. *N* = 25–28 worms/condition. ***G***, Number of GFP^+^ neurons detected in *pCRE::GFP* worms in a wild-type or *cmk-1* background treated with water and 1 h removal. **p < 0.005; ***p < 0.0005; ***p < 0.0001. Error bars are SEM. *N* = 27–31 worms/condition. For additional imaging data and controls, see Extended Data Figure 5-1*.*

Due to previous findings that *crh-1* is normally a transcriptional repressor of *glr-1* and our findings that losing *crh-1* is protective against *glr-1* downregulation during EtOH withdrawal, we next hypothesized that *crh-1*–dependent transcription is activated during withdrawal from chronic EtOH. To address this, we utilized a reporter of *crh-1* transcriptional activity (*pCRE::GFP*; Kimura et al., 2002) to track and measure CRH-1/CREB activity in neurons across all treatment conditions. Previous studies demonstrated that this reporter was responsive to starvation, leading to GFP expression in a specific population of neurons (the SIA; Suo et al., 2006). We repeated these conditions as a positive control for our ability to successfully detect changes in reporter activity, including the number of neurons detected and GFP intensities (Extended Data Fig. 5-1*A*–*C*). Next, we imaged *pCRE::GFP* worms exposed to water, EtOH, or 1 h EtOH withdrawal (Fig. 5*A*). We found that GFP intensity of both neurons and non-neuronal structures after 1 h withdrawal was similar to that of chronic EtOH treatment (Ext. Data Fig. 5-1*D*,*E*). However, the number of GFP+ neurons detected was higher in the withdrawal group versus the EtOH group (Fig. 5*E*), while the number and intensities of non-neuronal structures detected remained the same in both groups (Extended Data Fig. 5-1*E*,*F*). Overall, this suggests that neuronal CREB-mediated transcription is increased in neurons following 1 h EtOH withdrawal. Intriguingly, this broad activation is distinct from increases of CREB transcriptional activity in specific neurons that have been observed following starvation (Fig. 5*E*; Suo et al., 2006), as well as long-term memory training (Lakhina et al., 2015; Arey et al., 2018), further supporting that this CREB activation is part of an adaptive response in the nervous system.

10.1523/ENEURO.0430-25.2026.f5-1 Figure 5-1 A) Number of GFP+ neurons detected in *pCRE::GFP* untreated and positive controls (4h starved). ****p<.0001. Error bars are SEM. N=16-24 worms per condition. B) Neuronal GFP intensities of *pCRE::GFP* untreated and 4h starved controls, ****p<.0001 Error bars are SEM. N=28-30 ROIs per condition. C) Representative images of *pCRE::GFP* worms untreated vs. 4h starved controls. 100X objective. Scale bars = 10µM. D) Neuronal GFP intensities of *pCRE::GFP* worms treated with water, chronic EtOH, or 1h EtOH removal. N.s., not significant (p>.05), ***p<.0001. N=50-95 ROIs per condition. E) Non-neuronal GFP intensities in EtOH and 1h withdrawal treated *pCRE::GFP* worms. N.s., not significant (p>.05). Error bars are SEM. N=34-48 ROIs per condition. F) Number of non-neuronal GFP+ ROIs detected in EtOH-treated or withdrawal treated *pCRE::GFP* worms N.s., not significant (p>.05). Error bars are SEM.N=15-19 worms per condition. Download Figure 5-1, TIF file.

### JNK signaling mediates withdrawal-related changes in CREB transcriptional activity

Next, we wanted to try to uncover the molecular pathway underlying neuronal CREB-mediated transcription in neurons. CREB is known to be activated by kinases, and previous studies have shown that CREB activity can be induced as an adaptive response to changes in cell excitability, as well as oxidative stress generated by reactive oxygen species (ROS), both of which are linked to EtOH exposure or withdrawal (Lovinger, 1993; Hughes, 2009; Bansal et al., 2010; Zhong et al., 2012; Linhart et al., 2014; Peng et al., 2020; Ruiter-Lopez et al., 2025). Thus, we selected kinases upstream of CREB, JNK-1, and CMK-1, which are known to respond to these types of stressors (Ferrer et al., 2001; Feldmann et al., 2019; Zhu et al., 2019; Zhi et al., 2020; Mendelowitz, 2022). CMK-1 is orthologous to human calcium/calmodulin-dependent protein kinase I and IV (CAMKI, CAMKIV) because it acts both in the cell body and nucleus (Eto et al., 1999; Tokumitsu et al., 1999; Kimura et al., 2002). CMK-1 has been previously shown to regulate *glr-1* transcription in response to changes in neuronal activity (Moss et al., 2016), as well as negatively regulate excitotoxic stress via CREB (Feldmann et al., 2019; Mendelowitz, 2022), serving as a promising candidate for modulating *glr-1*/GLR-1 adaptations in EtOH withdrawal (Fig. 5*B*, right). On the other hand, c-Jun-N-terminal kinase (JNK-1) in the worm is orthologous to mammalian JNK proteins, enables JUN kinase activity, and is neuronally expressed (Bai et al., 2022). JNK-1 expression is induced in *C. elegans* neurons in response to EtOH exposure (Jee and Batsaikhan, 2024), and JNK signaling is also involved in cellular responses to excitotoxic and oxidative stress, including CREB activation (Ferrer et al., 2001; Zhu et al., 2019; Zhi et al., 2020; Bayón-Cordero et al., 2022; Zhang et al., 2025; Fig. 5*B*, left).

To determine if CMK-1 or JNK-1 were required for CREB-mediated transcription induction in EtOH removal, we crossed loss-of-function mutants *jnk-1(gk7)* and *cmk-1(oy21)* lines into the *pCRE::GFP* background. To our surprise, we found that loss of *jnk-1* completely abolished a significant increase in GFP signal following EtOH withdrawal (Fig. 5*C*,*F*), whereas loss of *cmk-1* only partially prevented GFP induction after EtOH withdrawal, although the number of GFP^+^ neurons were significantly lower in the *cmk-1* background versus the wild-type background (Fig. 5*D*,*G*). These data suggest that while *cmk-1* may partially regulate neuronal CREB-mediated transcription in EtOH withdrawal, *jnk-1* may be the central regulator of neuronal responses to EtOH withdrawal.

### JNK is required for *glr-1* downregulation and ITM behavior deficits in EtOH withdrawal

**Figure 6.**
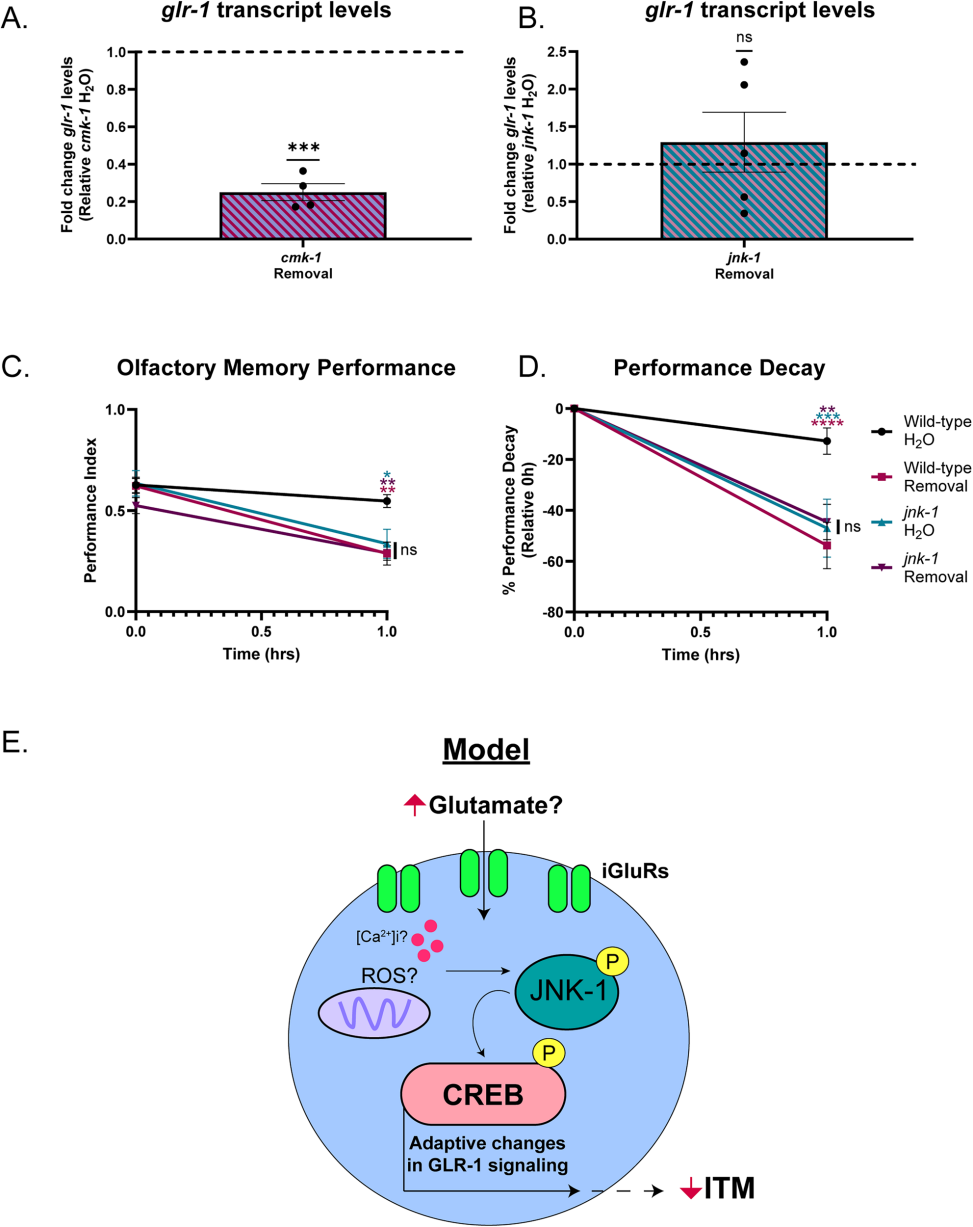
JNK-1 regulates *glr-1* expression and behavior deficits in EtOH withdrawal. ***A***, Relative fold change of *glr-1* transcript levels in *cmk-1* worms treated with 1 h removal versus water-treated *cmk-1* worms. ***p = 0.0005. Error bars are SEM. *N* = 4 biological replicates. Dashed lines indicated fold change of 1. ***B***, Relative fold change of *glr-1* transcript levels in *jnk-1* worms treated with 1 h removal versus water-treated *jnk-1* worms. N.s., not significant (*p* > 0.05). Error bars are SEM. *N* = 5 biological replicates. Dashed lines indicated fold change of 1. ***C***, Olfactory memory performance curves of wildtypes and *jnk-1* worms treated with water or 1 h removal. N.s., not significant (*p* > 0.05); *p < 0.05; **p < 0.005. Error bars are SEM. *N* = 15 plates per condition. ***D***, Olfactory memory performance decay curves of wild-type and *jnk-1* worms treated with water or 1 h removal. N.s., not significant (*p* > 0.05); **p < 0.005; ***p < 0.005; **p < 0.0005; ****p < 0.0001. Error bars are SEM. *N* = 15 plates per condition. ***E***, Tentative model of how JNK regulates neuroadaptive responses to EtOH withdrawal leading to CREB-dependent *glr-1* transcriptional repression.

Because we found that *pCRE::GFP* worms still show GFP induction in a loss of *cmk-1* background, we hypothesized that *glr-1* would still be downregulated in *cmk-1* mutants following EtOH withdrawal. Indeed, we found that *cmk-1* worms still displayed significant downregulation of *glr-1* following EtOH withdrawal (Fig. 6*A*). Given our qPCR and imaging data, we ruled out the central role of *cmk-1* in regulating CREB-dependent changes in *glr-1* transcription in EtOH withdrawal.

However, when we measured *glr-1* transcript levels in EtOH-removed *jnk-1* worms, we found that they maintained *glr-1* expression (Fig. 6*B*), further supporting that *jnk-1* is required for withdrawal-related *glr-1* downregulation. We reasoned that if JNK-1 was required for the transcriptional regulation of *glr-1* in the context of withdrawal, then loss of *jnk-1* would also occlude withdrawal-related memory deficits. Therefore, we tested olfactory associative memory in *jnk-1* worms versus wild types following treatment with water or EtOH withdrawal. We found that *jnk-1* worms treated with water perform significantly worse at the ITM time-point versus wild types, but their performance was not significantly lower than EtOH-removed wild types nor EtOH-removed *jnk-1* mutants (Fig. 6*C*), similar to what we had observed with *glr-1* mutants (Fig. 2*B*). In agreement, we found the memory decay between the learning and ITM time-point was significantly increased in magnitude in both water- and EtOH withdrawal-treated *jnk-1* worms (Fig. 6*D*). Importantly, neither the ITM performance nor the magnitude of memory decay were significantly different between water-treated and EtOH withdrawal-treated *jnk-1* worms. Overall, these data suggest that JNK-1 may play a central role in driving CREB-dependent *glr-1* downregulation and *glr-1*–regulated behavior deficits in EtOH withdrawal (Fig. 6*E*).

## Discussion

Animals have evolved to navigate the world and identify beneficial environments to promote their survival. The behaviors that animals use to respond to their environment can be modified by experience including contact with food, odorants, and other substances in the wild. Worms can encounter EtOH in the wild potentially through rotting fruit and plant matter (Félix and Duveau, 2012). EtOH is an energy source for worms (Patananan et al., 2015; Kaptan et al., 2020), and low concentrations extend lifespan in larvae (Yu et al., 2011; Castro et al., 2012). EtOH has molecularly conserved effects across phyla and can influence behaviors conserved in worms including state-dependent learning (Lindsay et al., 2022), sensitivity and tolerance (Morgan and Sedensky, 1995; Davies et al., 2003; Davies et al., 2004; Mitchell et al., 2007; Bettinger et al., 2012; Alaimo et al., 2012; Jee et al., 2013; Hawkins et al., 2015; Mathies et al., 2015; Johnson et al., 2017; Pandey et al., 2021; Guzman et al., 2022; Jee and Batsaikhan, 2024), EtOH preference (J. Lee et al., 2009), EtOH seeking (Salim et al., 2022), and nonassociative memory (Rose et al., 2022; Kokan et al., 2025). In this study, we leveraged *C. elegans* to model other behavioral phenotypes following EtOH withdrawal.

### EtOH withdrawal, but not chronic EtOH, disrupts ITM

We developed a new model to study how withdrawal from chronic EtOH impacts memory. We found that 20–24 h of EtOH treatment in Day 1 adult wild-type worms yielded similar internal EtOH concentrations as previously reported (Scott et al., 2017), and 1 h removal in Day 2 adult worms reduced EtOH to negligible levels prior to the olfactory associative memory assay. We found that EtOH withdrawal persistently represses ITM and temporarily inhibits navigation behaviors, which are regulated by *glr-1*. Specifically, navigation is restored after protracted EtOH withdrawal, whereas ITM is not, suggesting that memory is uniquely affected by EtOH withdrawal and precludes the possibility that neurons are irreversibly damaged. Supporting this, we found that ITM is rescued following reintroduction of a lower concentration of EtOH that yields detectable increases in internal EtOH levels, suggesting the ITM deficit depends on EtOH withdrawal. It is possible EtOH reintroduction was a contextual cue to promote memory, but because worms were not memory trained with butanone diluted in EtOH and we did not include EtOH in the training plates to avoid this confound, this is unlikely. Although we cannot fully model “withdrawal” in *C. elegans*, low-dose EtOH subverted the withdrawal-related ITM phenotype, similar to previous investigations of other *C. elegans* withdrawal behaviors (Mitchell et al., 2010; Scott et al., 2017) and somewhat analogous to withdrawal relief in humans and mammalian models (Heinz et al., 2003; Koob and Le Moal, 2005).

We found continuous EtOH exposure before behavioral training did not reduce ITM. While performance indices were significantly higher in EtOH-treated worms compared with water-treated worms, learning, and memory chemotaxis indices were not significantly different between treatments. Slight but insignificant differences in naive chemotaxis may have inflated the performance of the EtOH group. To circumvent the effects of naive chemotaxis on memory performance, we measured memory decay, which was not significantly different between treatments at any time-point. The resistance to memory deficits in the chronic EtOH group may reflect behavioral tolerance, a form of behavioral plasticity resulting in decreased responses over the course of drug exposure (Kalant, 1998; Pietrzykowski and Treistman, 2008). EtOH tolerance often involves molecular adaptations that function independently of altered pharmacokinetics (i.e., EtOH metabolism). These conserved mechanisms include but are not limited to genetics (Thiele et al., 1998; Scholz et al., 2000; Davies et al., 2003, 2004; Ghezzi et al., 2004; Scholz et al., 2005), epigenetic modifications (J. Wang et al., 2007; Ghezzi et al., 2017), lipid microenvironments (Crowley et al., 2003; Yuan et al., 2008; Bettinger et al., 2012), and alterations in membrane proteins (Davies et al., 2003; Kumar et al., 2003; Liang et al., 2007). Using the robust experimental tools available in *C. elegans*, it will be interesting to study mechanisms of EtOH tolerance in the context of memory deficits in the future. Fascinatingly, exposing worms to 400 mM EtOH during nonassociative memory training disrupted long-term memory (24 h memory) and blocked training-induced changes in GLR-1 puncta expression (Rose et al., 2022). This contrasts with our experiments, where worms received continuous exposure for 20–24 h prior to memory training and are trained sans EtOH exposure. It is possible that the duration of EtOH exposure during nonassociative memory training in the previous study was insufficient to recruit compensatory mechanisms that would permit normal long-term memory in pre-exposed worms. Together, these studies suggest that phases of EtOH exposure and withdrawal are discrete nervous system and behavioral states.

Previous work found EtOH withdrawal impairs food and odor race behaviors, and low concentrations of EtOH rescue these behaviors (Mitchell et al., 2010; Scott et al., 2017). Here, we identified a withdrawal-specific deficit in associative memory consolidation, increasing the repertoire of withdrawal-sensitive behaviors in *C. elegans*. It is possible we observed withdrawal-specific deficits after the worm has adapted to chronic EtOH due to the nervous system failing to readjust to EtOH withdrawal or rebounding in response to withdrawal. Adaptations in neuronal excitability and altered gene expression can occur between chronic EtOH and withdrawal (Lovinger and Roberto, 2025). Future investigations using *C. elegans* to assess rapid molecular adaptations in response to EtOH withdrawal will provide better understanding of conserved withdrawal-specific behaviors.

### EtOH withdrawal decreases *glr-1* expression and function

We found that EtOH withdrawal-related behavior deficits may involve *glr-1*, which regulates ITM in olfactory associative memory, and navigation behaviors. We found that EtOH withdrawal in wild-type animals phenocopies reduced GLR-1 function across both assays, and loss of *glr-1* occludes a further memory deficit induced by EtOH withdrawal. It was previously found that withdrawal from 6 h of 200–300 mM EtOH led to fewer reversals but a higher frequency of unaccompanied omega turns within 5 min of a food-race assay (Mitchell et al., 2010). This contrasts with our experimental setup, where worms are not racing but simply placed on a foodless plate and recorded within 12 min following transfer. Different EtOH concentrations, duration of exposure, and behavior-task specific contexts (presence or absence of food) may explain this discrepancy. Overall, our data suggest that EtOH withdrawal-related phenotypes point to *glr-1* downregulation.

Interestingly, we found that navigation behaviors began to restore over time, following 2 h of EtOH withdrawal. Because navigation behaviors are restored and baseline chemosensation is not significantly impaired in EtOH withdrawal, this suggests that our EtOH exposure and withdrawal paradigm does not irreparably damage nervous system function. ITM performance, however, was still poor following 2 h of EtOH withdrawal. The persistent memory deficit may be due to the timing of *glr-1* downregulation within the context of the olfactory associative memory assay. It is possible that *glr-1* downregulation occurs prior to memory training (within 1 h of EtOH withdrawal), negating memory formation. Thus, while the return of navigation behaviors suggests that *glr-1* expression or function is eventually restored, this occurs too far removed from memory training to permit memory formation. However, 2 h withdrawal also enhanced forgetting, suggesting additional adaptations occur that accelerate forgetting in protracted withdrawal, either through *glr-1* or other regulators of forgetting in parallel (Liu et al., 2022).

To determine the relationship between *glr-1*/GLR-1 expression and behavior deficits in EtOH withdrawal, we measured transcript levels of *glr-1* and GLR-1 abundance in the VNC following chronic EtOH or 1 h EtOH withdrawal. Following chronic EtOH, *glr-1* transcript levels were similar to water-treated controls, whereas the withdrawn group showed significant *glr-1* downregulation compared with chronic EtOH and water-treated worms. When we measured GLR-1 in the VNC, we found that chronic EtOH increased puncta intensity and size, indicating increased GLR-1 expression. However, after EtOH withdrawal, fluorescence intensity significantly decreased compared with chronic EtOH treatment. These data suggest *glr-1*/GLR-1 is dynamically regulated to respond to first chronic EtOH treatment and later sudden EtOH withdrawal. It is possible that GLR-1 accumulates in the VNC to adapt to chronic EtOH but normalizes once EtOH is abruptly removed, coinciding with reducing *glr-1* transcription. Additionally, downregulation of *glr-1*/GLR-1 in EtOH withdrawal may prevent harmful increases in glutamatergic signaling. Decreased in *glr-*1/GLR-1 expression in early EtOH withdrawal may reflect a “trade-off,” protecting neurons at the expense of memory consolidation and navigation behaviors. Investigating GLR-1 regulatory mechanisms in chronic EtOH and withdrawal, including localization, transport, or internalization, is an exciting avenue for future study. While we did not measure the effects on EtOH reintroduction, which can abrogate the ITM deficits we observe after withdrawal, on *glr-1* mRNA levels or synaptic GLR expression here, it will be exciting in the future to determine if EtOH reintroduction can reverse molecular adaptations in *glr-1*/GLR-1 following EtOH withdrawal.

There are conflicting reports in other models, including mammalian in vitro, in vivo, and human studies as to how AMPARs and glutamate signaling are regulated in withdrawal, although dynamic modulation of AMPAR expression and glutamate signaling is commonly observed. Some studies report increased AMPAR levels and signaling (Christian et al., 2012; Wang et al., 2012, 2015; Gerace et al., 2019, 2021), decreased AMPA expression, neuronal excitability, or glutamate levels (Ulrichsen et al., 1996; Stephens et al., 2005; Francesconi et al., 2009; Kryger and Wilce, 2010; Thoma et al., 2011; Mon et al., 2012; Pickering et al., 2015; Das et al., 2016) or no change in AMPAR levels (Trevisan et al., 1994; Freund and Anderson, 1996; Rudolph et al., 1997; Ferreira et al., 2001; Williams et al., 2018). Heavy drinking is associated with lower glutamate levels in the brain (Ende et al., 2013; Prisciandaro et al., 2016; Prisciandaro et al., 2019) although these changes may depend on severity of alcohol-use disorder (Ende et al., 2013; Prisciandaro et al., 2020). Altered glutamate signaling and neuronal excitability are linked to EtOH withdrawal-dependent behaviors including anxiety and seizures (Krupitsky et al., 2007; Läck et al., 2007; Brousse et al., 2012; Becker and Mulholland, 2014), whereas lower AMPAR levels are linked to lower neuronal excitability, LTP capability, and memory. Possible explanations for these discrepancies include withdrawal length, EtOH exposure paradigm, brain region, and behavioral investigations, highlighting the difficulty in fully unraveling how withdrawal impacts glutamate receptor signaling and behavior. However, our study is consistent with reports of adaptive changes in glutamate signaling and AMPAR expression across EtOH exposure and withdrawal.

### The role of CREB in *glr-1*/AMPAR regulation in EtOH withdrawal

Prolonged EtOH exposure in *C. elegans* impacts gene expression that regulates behaviors in withdrawal (Mathies et al., 2026). We found loss of master transcriptional regulator *crh-1*/CREB prevents *glr-1* downregulation and some *glr-1*-dependent behavior deficits in withdrawal. Although *crh-1* mutants alone did not have higher *glr-1* transcript levels, they performed significantly more reversals and omega turns compared with wild types, which is associated with increased glutamate receptor function (Zheng et al., 1999). Because CREB regulates synaptic protein expression, increased GLR-1 protein expression or synaptic function could explain *crh-1* mutant navigation rather than mRNA levels. Critically, EtOH withdrawal did not repress *glr-1* levels nor navigation behaviors in *crh-1* worms. Furthermore, we found CREB-dependent transcription increased in neurons following EtOH withdrawal. This broad and likely adaptive change in CREB-dependent transcription across the nervous system, which is distinct from neuron specific increases following memory training (Lakhina et al., 2015; Arey et al., 2018), may lead to global *glr-1*/GLR-1 expression changes, ultimately affecting behavior. Normally, CREB is required for long-term memory and is not required for transcription-independent memory across species (Frank and Greenberg, 1994; Carew, 1996; Lamprecht et al., 1997; Kauffman et al., 2010; Lakhina et al., 2015; Arey et al., 2018). In long-term memory, CREB's activation and interaction with coactivators in specific neurons leads to persistent changes affecting gene expression and synapse dynamics following neuronal activity and associative learning (Deisseroth et al., 1996; Kandel, 2012; Nonaka et al., 2014; Hirano et al., 2016; Parra-Damas et al., 2017).

We hypothesize that EtOH withdrawal-related CREB activity in more neurons could result in aberrantly upregulated plasticity-related genes, which could alter synaptic connectivity and disrupt behavior. Interestingly, previous studies found increased CREB expression or activation after withdrawal from EtOH (Pandey et al., 2001; Bison and Crews, 2003; J. Wang et al., 2007; Shibasaki et al., 2011; Freeman et al., 2013; Kurokawa et al., 2013; Qiao et al., 2018), and other drugs of abuse (Fijał et al., 2015; Navarro-Zaragoza et al., 2017). CREB is linked to withdrawal-related behaviors including anxiety, EtOH consumption, preference, tolerance, and spatial memory deficits (Pandey et al., 1999, 2004; J. Wang et al., 2007; Kurokawa et al., 2013; Dominguez et al., 2016; Qiao et al., 2018). The complexity of the relationship between CREB and EtOH may be due to similar discrepancies described above for AMPARs. Overall, our findings agree with the literature that EtOH exposure and withdrawal recruit CREB-dependent transcription, with additional findings that loss of CREB protects *glr-1*–regulated navigation behaviors in EtOH withdrawal.

### JNK-1 is required for CREB-dependent transcription, *glr-1* expression, and behavior deficits in EtOH withdrawal

We next wanted to understand the molecular pathway associated with CREB-mediated transcription and related behavioral phenotypes in EtOH withdrawal. We found that only one of our candidate kinases, JNK-1, was involved, which was somewhat surprising given the other candidate CMK-1's established role in responding to stress-induced changes in glutamate signaling (Feldmann et al., 2019; Mendelowitz, 2022).

Conversely, loss of *jnk-1* occluded phenotypes associated with EtOH withdrawal: ITM deficits, *glr-1* downregulation, and CREB-dependent transcription. These results suggest that JNK mediates CREB-dependent changes in *glr-1* transcription and behavior in EtOH withdrawal. JNK kinases belong to the family of mitogen-activated protein kinases (MAPKs), which respond to stressful stimuli, including EtOH, oxidative stress, or changes in glutamate signaling (Kim and Choi, 2010; Vieira et al., 2010; Jee and Batsaikhan, 2024; Zhang et al., 2025). JNK becomes activated through a phosphorylation cascade of upstream MAP kinases bound by JIP scaffold proteins (Tournier et al., 1997; Sundaram, 2006; Haeusgen et al., 2011). Orthologs of this cascade have been identified in *C. elegans* (Sagasti et al., 2001; Sundaram, 2006; Wolf et al., 2008; Pastuhov et al., 2015). Once JNK is phosphorylated, it can activate substrates including transcription factors to drive cell survival or death in response to stress. Additionally, JNK regulates memory and synaptic AMPAR expression (Bevilaqua et al., 2003; Zhu et al., 2005; Kim et al., 2007; Thomas et al., 2008; Sherrin et al., 2010; Lakhina et al., 2015; Hollos et al., 2020). Because of JNK's known role in regulating memory, it was unsurprising that water-treated *jnk-1* mutants had ITM deficits. However, the lack of a further deficit from EtOH withdrawal in *jnk-1* mutants suggests JNK regulates this phenotype in withdrawal. Because GLR-1/AMPAR trafficking is regulated by JNK and MAPK signaling (Zhu et al., 2002; Zhu et al., 2005; Thomas et al., 2008; Chen et al., 2014; Hoerndli et al., 2022), it is possible that losing JNK-1 prevents GLR-1 accumulation in chronic EtOH, negating CREB-dependent transcriptional adaptations. Future studies should investigate the role of JNK in GLR-1 localization and trafficking across EtOH exposure conditions and identify upstream components involved in this pathway.

How might EtOH withdrawal recruit JNK signaling in *C. elegans*? Chronic EtOH and withdrawal create conditions that activate JNK, including adaptive changes in cell excitability (via altered glutamate signaling), and oxidative stress (Dugan and Choi, 1994; Huang et al., 2009; Zhong et al., 2012; Clergue-Duval et al., 2022). Briefly, oxidative stress occurs when ROS outweigh the antioxidant capacity of a cell. ROS or oxidative stress can be generated from EtOH metabolism itself, including in the nervous system (Zakhari, 2006; Zimatkin et al., 2006; Reddy et al., 2013; Wang et al., 2013), or by mitochondrial dysfunction in response to EtOH-induced adaptive changes in glutamate signaling and intracellular calcium levels (Reynolds and Hastings, 1995; Reynolds, 1999; Jung and Metzger, 2010; Jakaria et al., 2018; Clergue-Duval et al., 2022). ROS regulates GLR-1 trafficking as well (Doser et al., 2020, 2024) and could explain altered GLR-1 expression in our study. Glutamate and ROS can also activate JNK and CREB (Liu et al., 2002; Zou and Crews, 2006; B. Lee et al., 2009; Shah et al., 2014; Bayón-Cordero et al., 2022; Németh et al., 2025), and stress promotes CREB activation via JNK (Schwarzschild et al.1997; B. Lee et al., 2009; Shah et al., 2014). While JNK can drive stress-induced apoptosis (Yang et al., 1997; Liu et al., 2002; Nadeau et al., 2009), it can also protect cells, perhaps by sensing and transmitting stress signals to distal targets to promote survival (Lindwall and Kanje, 2005; Pastuhov et al., 2015; Santabárbara-Ruiz et al., 2015; Zhi et al., 2020; Yin et al., 2022; Quinn et al., 2025). The manner and duration of stress induction, JNK activation, or downstream JNK targets in these studies may explain the dual role of JNK in cell death and protection.

Finally, what are the potential CREB targets that downregulate *glr-1*? There is not a known CRH-1/CREB binding site on *glr-1*, suggesting its downregulation occurs indirectly. While CREB becomes activated in response to glutamate signaling or oxidative stress, few CREB targets are known in these contexts. For example, CMK-CREB signaling drove transcription of ion channels *kqt-1* and *clh-4* in GLR-1^+^ neurons and attenuated excitotoxic necrosis in larval *C. elegans* (Mendelowitz, 2022). Additionally, antiapoptotic protein *bcl-2* was upregulated via CREB in neurons or cell culture models following excitotoxic or oxidative stress (Cheung et al., 2000; Kitagawa, 2007; Kim et al., 2013; Zhi et al., 2020). However, targets of JNK and CREB in EtOH withdrawal are unknown.

Based on our data and these previous studies, we hypothesize this JNK–CREB pathway is an adaptive response to stress via regulating GLR-1 signaling. JNK may serve as the sensor for these changes, initiating a CREB-dependent broad neuroadaptive response, decreasing *glr-1* production and repressing *glr-1*-regulated behaviors. Alternatively, JNK may regulate GLR-1 expression during chronic EtOH, triggering a compensatory CREB-dependent decrease in *glr-1* production. Regardless, this pathway may protect neurons at the cost of *glr-1*–dependent behaviors, including memory.

Overall, this study suggests that EtOH withdrawal produces potentially adaptive postsynaptic *glr-1*/GLR-1 expression changes via JNK and CREB-dependent transcription, driving deficits in positive olfactory associative memory. Future studies should test whether JNK regulates synaptic GLR dynamics in EtOH exposure, identify transcriptional targets of this JNK–CREB signaling axis, and characterize additional transcription factors possibly involved in behavior deficits in EtOH withdrawal.

## Synthesis

Reviewing Editor: Jibran Khokhar, Western University Department of Anatomy and Cell Biology Decisions are customarily a result of the Reviewing Editor and the peer reviewers coming together and discussing their recommendations until a consensus is reached. When revisions are invited, a fact-based synthesis statement explaining their decision and outlining what is needed to prepare a revision will be listed below. The following reviewer(s) agreed to reveal their identity: Jill Bettinger, Jacqueline Rose.

The manuscript presents an interesting and potentially important finding that withdrawal from extended ethanol exposure selectively disrupts intermediate-term memory and other glutamate-dependent behaviors in C. elegans, accompanied by reduced glr-1 transcript levels. However, significant conceptual, methodological, and interpretational weaknesses limit confidence in the conclusions, particularly regarding the definition of ethanol withdrawal, distinction between learning and memory deficits, and the strength of evidence supporting a regulated glr-1-dependent mechanism.

Major concerns

Why one hour of ethanol withdrawal is considered behaviorally meaningful is not justified, and it is unclear whether this time point represents acute withdrawal or an early abstinence-like state; additional withdrawal and post-conditioning time points, such as two hours after conditioning, should be tested behaviorally and molecularly.

A defining feature of withdrawal is behavioral compensation or rescue upon reexposure to ethanol, which was not tested and is necessary to support a withdrawal-based interpretation.

The observed behavioral deficits could reflect nonspecific neuronal damage rather than regulated neuroadaptation; it should be demonstrated whether the phenotypes are transient or reversible to rule out ethanol-induced toxicity.

Behavioral analyses conflate learning and memory expression; comparisons should focus on within-genotype changes over time rather than between-genotype comparisons at a single time point.

In Figure 2, the appropriate comparison is the difference in chemotaxis index between 0 and 1 hour within each genotype, not mutant versus wild-type at one hour.

In Figures 2C and 2D, glr-1 animals show little decline from 0 to 1 hour, resembling wild-type behavior in Figures 2A and 2B and Figure 1C, suggesting intact intermediate-term memory rather than impairment.

It is unclear whether glr-1 mutants in Figures 2A and 2B show a statistically significant difference between 0 and 1 hour time points.

The ptp-3(mu256) strain appears to show impaired acquisition rather than a specific intermediate-term memory deficit, making it an inappropriate positive control.

According to Pierce et al. (2022), deficits in GLR-1 dynamics and learning and memory are associated with ptp-3(ok244), not ptp-3(mu256), raising concerns about strain selection and interpretation.

It is unclear whether animals in the "EtOH" condition were tested during acute withdrawal or on ethanol-containing assay plates; if the latter, a critical control of water-treated animals tested on ethanol is missing. If they are in acute withdrawal, the interpretations of the results should be modified accordingly after thinking deeply about what the behavior is telling us.

Ethanol concentration is only mentioned in a figure legend and not clearly described in the Methods or Results, and justification for the chosen concentration is lacking.

The use of blood alcohol concentration terminology is incorrect for worms, and tissue ethanol levels were not directly measured.

Because Scott et al. (2017) used first-day adults and this study used second-day adults, tissue ethanol concentrations cannot be assumed and should be directly measured both during ethanol exposure and after withdrawal.

The finding of increased ethanol chemotaxis in Extended Data Figure 1-1 could reflect a general increase in chemotaxis to weakly attractive odorants rather than an ethanol-specific effect and should be controlled accordingly.

Reduced glr-1 transcript levels measured by qPCR are insufficient to support a functional mechanism; protein-level validation such as Western blotting is required.

Given extensive post-translational regulation of GLR-1 receptors, transcript reductions do not necessarily predict functional synaptic changes within a one-hour withdrawal window.

Imaging of GLR-1 synaptic puncta using fluorescent reporters should be performed across multiple time points to assess receptor localization and abundance.

The statement that ethanol withdrawal phenocopies loss of GLR-1 function is not supported for memory outcomes, as glr-1 mutants under withdrawal conditions show higher memory indices than wild-type withdrawal animals and these conditions are not directly compared.

Investigation of crh-1 as an upstream regulator is inconclusive, as decreases in glr-1 transcripts are also observed in daf-8 and wild-type animals.

crh-1 transcriptional reporter activity increases broadly in response to starvation, ethanol exposure, and ethanol withdrawal, indicating low specificity and limiting mechanistic conclusions.

Overall, while glr-1 may contribute to ethanol withdrawal effects, the behavioral characterization is incomplete and the proposed molecular mechanism is not sufficiently established.

Minor concerns

Line 63 describes ethanol research in worms as "recent," which is inaccurate given the long history of work in this field.

Line 67 contains a cessation/cessation typo.

Lines 88-89 should explicitly define the time interval from learning used to classify intermediate-term memory.

Lines 91-92 contain confusing wording regarding whether the molecular pathway implicating crh-1 is also downregulated.

Line 138 does not specify the concentration of sodium azide or whether butanone was diluted in ethanol or water.

Extended Data Figure 1C and 1D do not clearly specify which odorant was used for chemotaxis.

Line 163 incorrectly implies that memory is absent at two hours post-learning, despite evidence from Stein and Murphy (2014) showing retained conditioned preference at that time point.

Line 200 reports imaging at 8-bit depth, which is insufficient for quantitative fluorescence analysis; a minimum of 12-bit depth is standard.

Line 237 should specify that training was massed rather than spaced.

Lines 267-268 should explicitly state that butanone was the chemosensory cue.

The Methods do not state that strains were not outcrossed, which is now standard practice and should be disclosed.

Non-parametric statistics used for reversals and omega turns in Figure 5 are not described in the Methods.

The rationale for using Mann-Whitney U tests for imaging data is not explained.

Figure legends contain dense statistical reporting that would be better summarized or moved to a table.

Figures are cited out of order, with Figure 1B cited before Figure 1A.

Figure 1 legend lettering does not align with the figure panels, including duplicate or misassigned labels.

Extended Figure 3D and 3E are reversed.

Extended Figure 2A does not support the claim of poorer intermediate-term memory in ptp-3 mutants when acquisition levels are considered.

Witvliet et al. (2021) would be more appropriate as support for defined circuits rather than associative memory.

Rose et al. (2003) is not an example of associative memory and should be removed or replaced.

Rose et al. (2022) citation should be corrected and added to the reference list.

Pierce et al. (2022) citation and reference should be updated.

Popova reference requires correction.

Typos or erroneous entries for Carew et al. (1996), Mon et al. (2012), and Thiel et al. (1998) should be deleted.

Ghezzi and Atkinson (2016) reference contains a typo.

The Gharat reference should include date of online publication, volume information, and DOI.
