# Supplementary figures and images for: Altered glr-1 Expression after EtOH Withdrawal Is Linked to Associative Behavior Deficits in C. elegans
Source: eNeuro. 2026 Jul 28;13(7):ENEURO.0430-25.2026. doi: 10.1523/ENEURO.0430-25.2026 (PMC13423511; doi:10.1523/ENEURO.0430-25.2026)

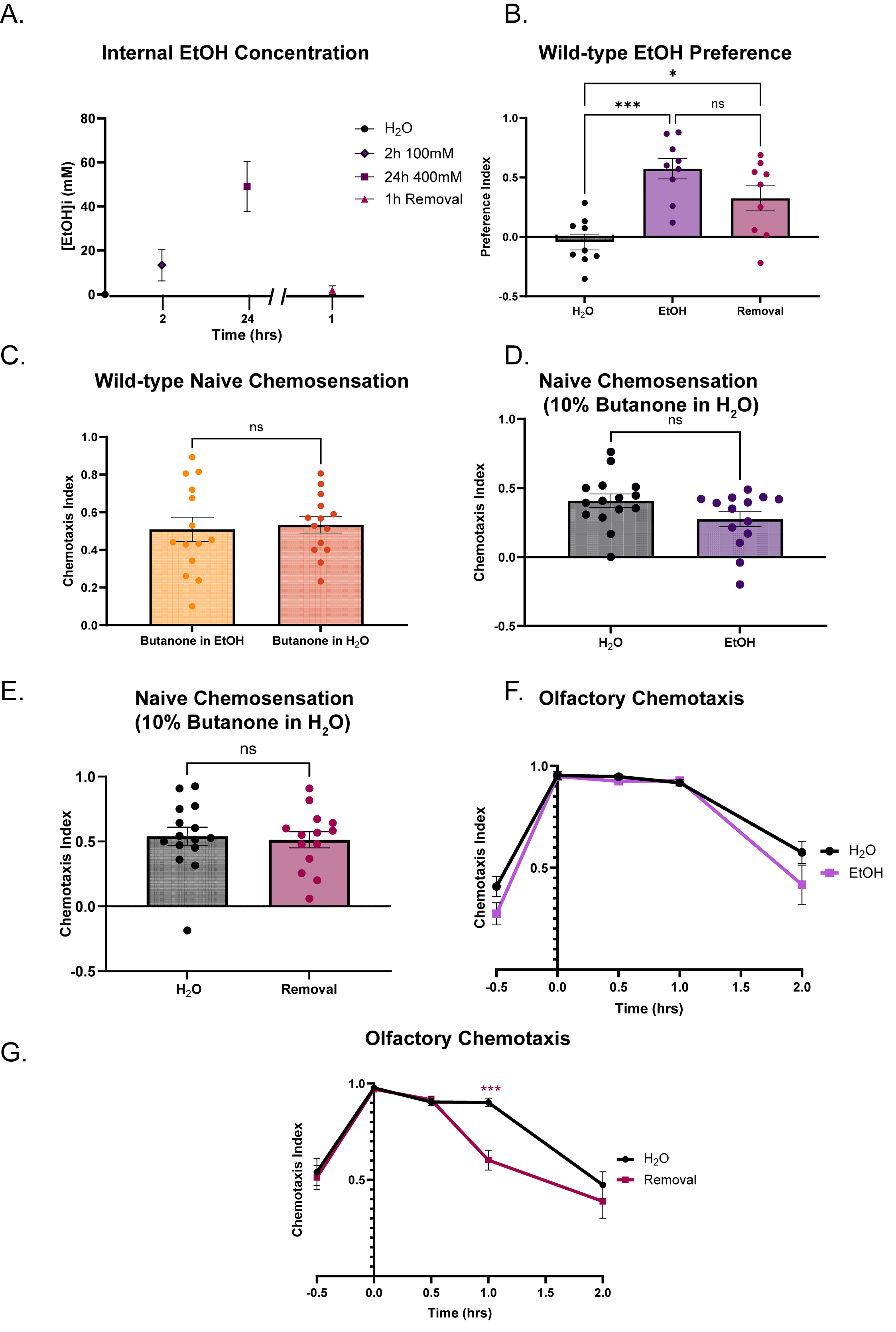

Supplement: Figure 1-1 — A) Internal EtOH concentrations of day 2 adult wild-type worms across different exposure time-points, including 1h of EtOH withdrawal N = 200 worms per condition, 3-4 biological replicates per condition. B) EtOH preference indices across treatment conditions in wild-type worms. N.s., not significant (p>.05), *p<.05, ***p<.005. Error bars are SEM. N = 9 plates per condition. C) Naïve chemotaxis indices of wild-type worms with the test odorant (10% butanone) diluted in EtOH vs. water. N.s, not significant (p>.05). Error bars are SEM. N=14 plates per condition. D) Naïve chemotaxis indices of water-treated and chronic EtOH-treated wild-types to 10% butanone diluted in water. N.s., not significant (p>.05). Error bars are SEM. N=14-15 plates per condition. E) Naïve chemotaxis indices of water-treated and removal-treated wild-types to 10% butanone diluted in water. N.s., not significant (p>.05). Error bars are SEM. N=14-15 plates per condition. F) Olfactory chemotaxis curve across all time-points in water-treated and EtOH-treated wildtype worms. N.s., not significant (p>.05). Error bars are SEM. G) Olfactory chemotaxis curve across all time-points in water-treated and removal-treated wildtype worms. ***p<.0005. Error bars are SEM. N=14-15 plates per condition. Download Figure 1-1, TIF file. [file eneuro-13-ENEURO.0430-25.2026-s002.tif]

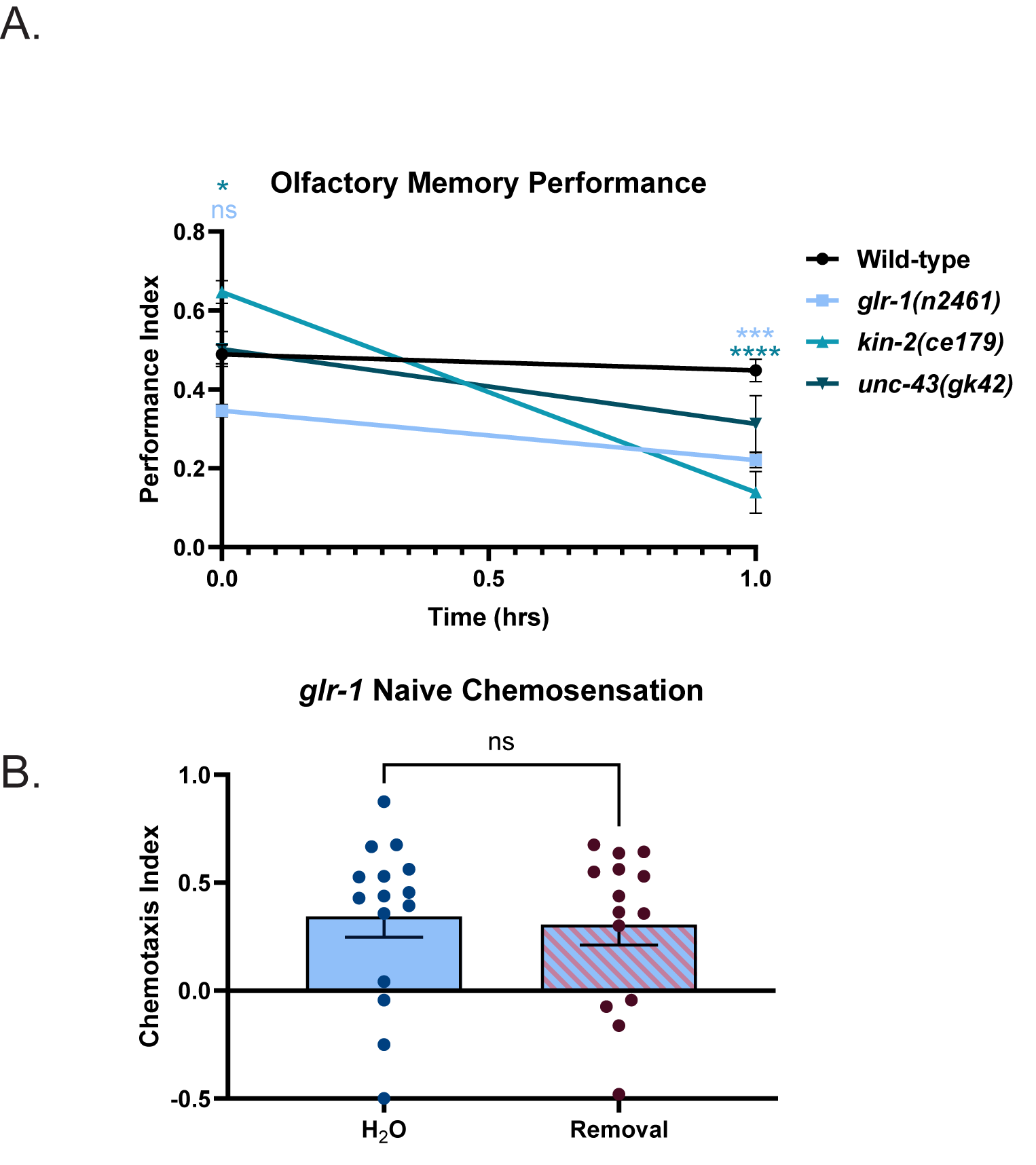

Supplement: Figure 2-1 — A) Olfactory memory performance across T0 and T60 time-points in wildtype, glr-1, kin-2, and unc-43 mutant worms. N.s., not significant (p>.05), *p<.05, ***p<.0007, ****p<.0001. Error bars are SEM. N=15 plates per condition. B) Naïve chemotaxis in glr-1 worms treated with water or 1h removal. N.s., not significant (p>.05). Error bars are SEM. N=14-15 plates per condition. Download Figure 2-1, TIF file. [file eneuro-13-ENEURO.0430-25.2026-s003.tif]

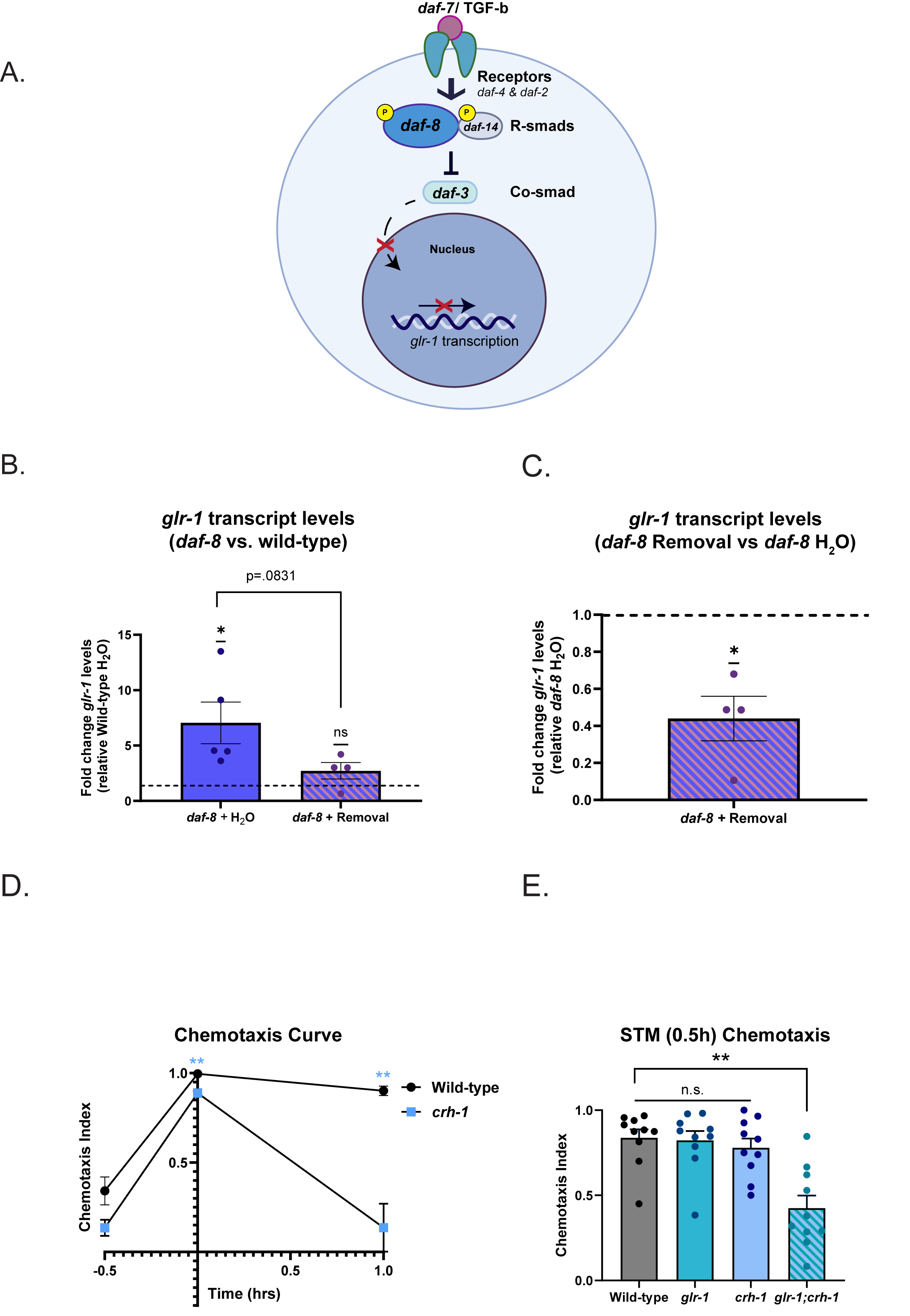

Supplement: Figure 4-1 — A) Pathway describing glr-1 transcriptional regulation via DAF-7/TGF-β. B) Relative fold change of glr-1 mRNA levels in daf-8 background relative to wild-type controls N.s. not significant (p>.05), *p<.05. Error bars are SEM. N=4-5 Biological replicates. Dashed lines indicated fold change of 1. C) Relative fold change of glr-1 levels in daf-8 treated with 1h removal vs. water-treated daf-8 worms. *p<.05. Error bars are SEM. N=4 Biological replicates. Dashed lines indicated fold change of 1. D) Olfactory chemotaxis curves of wildtypes and crh-1(tz2) worms. **p<.005. Error bars are SEM. N=9-10 plates per condition. E) Olfactory chemotaxis index of wildtypes, glr-1(n2461), crh-1(tz2), and glr-1;crh-1 worms at T30 (STM) time-point. N.s., not significant (p>.05), **p<.005. Error bars are SEM. N=10 plates per condition. Download Figure 4-1, TIF file. [file eneuro-13-ENEURO.0430-25.2026-s004.tif]

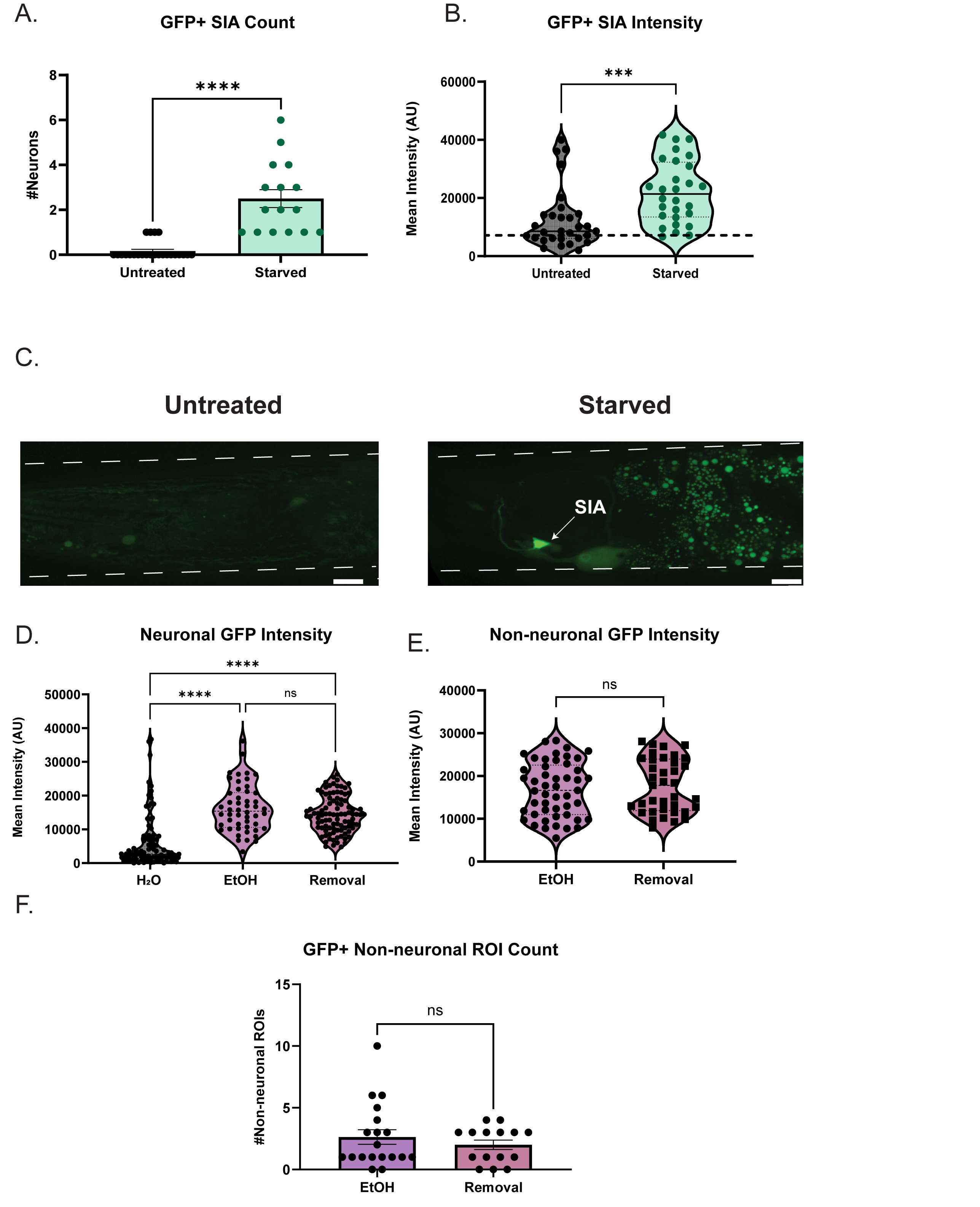

Supplement: Figure 5-1 — A) Number of GFP+ neurons detected in pCRE::GFP untreated and positive controls (4h starved). ****p<.0001. Error bars are SEM. N=16-24 worms per condition. B) Neuronal GFP intensities of pCRE::GFP untreated and 4h starved controls, ****p<.0001 Error bars are SEM. N=28-30 ROIs per condition. C) Representative images of pCRE::GFP worms untreated vs. 4h starved controls. 100X objective. Scale bars = 10µM. D) Neuronal GFP intensities of pCRE::GFP worms treated with water, chronic EtOH, or 1h EtOH removal. N.s., not significant (p>.05), ***p<.0001. N=50-95 ROIs per condition. E) Non-neuronal GFP intensities in EtOH and 1h withdrawal treated pCRE::GFP worms. N.s., not significant (p>.05). Error bars are SEM. N=34-48 ROIs per condition. F) Number of non-neuronal GFP+ ROIs detected in EtOH-treated or withdrawal treated pCRE::GFP worms N.s., not significant (p>.05). Error bars are SEM.N=15-19 worms per condition. Download Figure 5-1, TIF file. [file eneuro-13-ENEURO.0430-25.2026-s005.tif]
